# Simple gene signature to assess murine fibroblast polarization

**DOI:** 10.1038/s41598-022-15640-6

**Published:** 2022-07-11

**Authors:** Emmanuel Ledoult, Manel Jendoubi, Aurore Collet, Thomas Guerrier, Alexis Largy, Silvia Speca, Solange Vivier, Fabrice Bray, Martin Figeac, Eric Hachulla, Myriam Labalette, Frédéric Leprêtre, Shéhérazade Sebda, Sébastien Sanges, Christian Rolando, Vincent Sobanski, Sylvain Dubucquoi, David Launay

**Affiliations:** 1 Inserm, U1286, 4Ème Étage Centre, Place Verdun, 59000 Lille, France; 2 INFINITE - Institute for Translational Research in Inflammation, Univ. Lille, 59000 Lille, France; 3 Service de Médecine Interne et d’Immunologie Clinique, Centre de Référence Des Maladies Auto-Immunes et Systémiques Rares du Nord et Nord-Ouest de France (CeRAINO), CHU Lille, 59000 Lille, France; 4 Laboratoire d’Immunologie, Pole Biologie et d’Anatomopathologie, CHU Lille, 59000 Lille, France; 5 CNRS, UAR 3290 - MSAP - Miniaturisation Pour La Synthèse, Univ. Lille, l’Analyse et la Protéomique, 59000 Lille, France; 6 CNRS, Inserm, CHU Lille, Institut Pasteur de Lille, US 41 - UMS 2014 - PLBS, Univ. Lille, 59000 Lille, France; 7 Shrieking Sixties, 1-3 Allée Lavoisier, 59650 Villeneuve-d’Ascq, France; 8 Institut Universitaire de France (IUF), 75005 Paris, France

**Keywords:** Immunology, Biomarkers, Diseases, Medical research, Pathogenesis, Rheumatology

## Abstract

We provide an original multi-stage approach identifying a gene signature to assess murine fibroblast polarization. Prototypic polarizations (inflammatory/fibrotic) were induced by seeded mouse embryonic fibroblasts (MEFs) with TNFα or TGFß1, respectively. The transcriptomic and proteomic profiles were obtained by RNA microarray and LC-MS/MS. Gene Ontology and pathways analysis were performed among the differentially expressed genes (DEGs) and proteins (DEPs). Balb/c mice underwent daily intradermal injections of HOCl (or PBS) as an experimental murine model of inflammation-mediated fibrosis in a time-dependent manner. As results, 1456 and 2215 DEGs, and 289 and 233 DEPs were respectively found in MEFs in response to TNFα or TGFß1, respectively*.* Among the most significant pathways, we combined 26 representative genes to encompass the proinflammatory and profibrotic polarizations of fibroblasts. Based on principal component analysis, this signature deciphered baseline state, proinflammatory polarization, and profibrotic polarization as accurately as RNA microarray and LC-MS/MS did. Then, we assessed the gene signature on dermal fibroblasts isolated from the experimental murine model. We observed a proinflammatory polarization at day 7, and a mixture of a proinflammatory and profibrotic polarizations at day 42 in line with histological findings. Our approach provides a small-size and convenient gene signature to assess murine fibroblast polarization.

## Introduction

Fibroblasts play an important role in the regulation of the extracellular matrix (ECM) biosynthesis, crosslinking and degradation^1^. Fibroblasts are also involved in the acute inflammatory response to injury and infection^2^. Indeed, the recruitment and activation of inflammatory cells are the consequence of many signals including factors released by fibroblasts activated by damage- and pathogen-associated molecular patterns^3^. TNFα signaling during inflammation induces in turn a proinflammatory polarization of fibroblasts specialized in chemo-attraction of immune cells and degradation of ECM. In response to tissue injury, some fibroblasts also acquire a myofibroblast phenotype, characterized by the expression of contractile proteins, and enhance ECM biosynthesis^2^. This matrix producer specialization is tightly controlled in normal conditions while in fibrotic diseases fibroblasts remain activated. The TGFβ/Smad pathway plays a central role in the profibrotic polarization of fibroblasts^4,5^, which is characterized by an excessive biosynthesis of ECM, an over-crosslinking, and a decrease of ECM degradation processes disrupting the tissue architecture and leading to fibrosis^6–8^. In an experimental murine model of inflammation-mediated fibrosis induced in a time-dependent manner, proinflammatory and profibrotic fibroblasts coexisted. The proinflammatory polarization predominated at early stage, while the profibrotic polarization predominated at the late stage^9,10^.

The profibrotic polarization of fibroblasts is usually based on the evaluation of the gene expression of *Col1a1* and *Acta2*, as markers of myofibroblasts, a central subpopulation of fibroblasts involved in ECM-overproduction^4,11^. Yet, none is specific on its own. *Col1a1* is both upregulated in the inflammatory stage of the healing process and in fibrosis^9,10,12^. Several recent studies suggest that ECM overproduction is not limited to *Acta2* + myofibroblasts and that *Acta2* should not be used as a single marker of myofibroblastic transformation^9,10,13,14^. Interpretation based on a limited number of genes can therefore be over-simplistic and lead to a misinterpretation of the polarization of fibroblasts. Deeper approaches like single-cell RNA sequencing have highlighted the heterogeneity of fibroblasts in models of wound healing and of fibrosis identifying new potential markers of fibroblast polarizations^12,14,15^. For example, it has been reported that other isoforms than *Col1a1* were more expressed especially within ECM-producing fibroblasts (i.e. *Col13a* +) and inflammatory fibroblasts (i.e. *Col5a3* + , *Col14a* +)^14^. Yet, these omics tools are time- and cost-consuming making them not suitable to explore the polarization of fibroblasts in routine.

Defining the polarization of fibroblasts and its evolution during time is very important to better understand the kinetic and pathophysiology of inflammation and fibrosis and to assess the impact of drugs, especially in animal models. To be more complete than overly simplistic single markers and more accessible than full (single cell) transcriptomics, we designed this study to find an accessible and accurate gene signature reflecting the polarization of fibroblasts. Firstly, we induced in vitro proinflammatory or profibrotic polarizations by treating Mouse Embryonic Fibroblasts (MEFs) with proinflammatory or profibrotic cytokines. Secondly, we selected polarization-representative genes using a combined analysis of the transcriptome assessed by RNA microarrays and of the proteome assessed by mass spectrometry (LC–MS/MS). Finally, we applied this gene signature at the inflammatory stage and at the fibrotic stage on dermal mouse primary fibroblasts (dMPF) isolated from an experimental murine model of inflammation-mediated fibrosis in a time-dependent manner^16–19^.

## Results

### Mouse embryonic fibroblasts treated by TNFα or TGFß1 are representative of proinflammatory or profibrotic polarizations of fibroblasts

**Figure 1:**
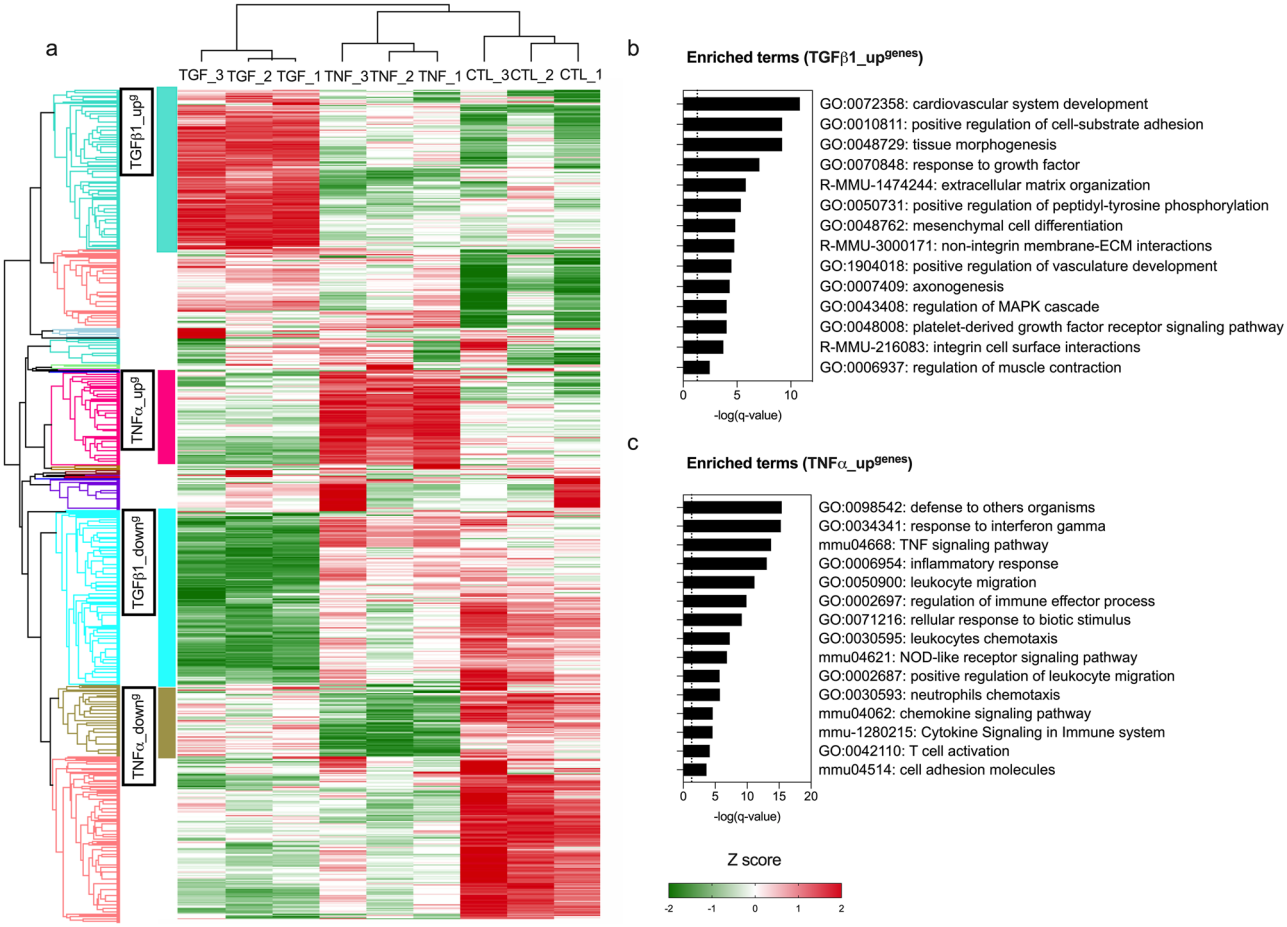
Transcript expression profiles of control, TGFß1-treated and TNFα-treated MEFs using RNA microarrays. (**a**) Heatmap summarizing DEGs in TGFß1- (5 ng/ml) or TNFα- (10 ng/ml) treated MEFs for 24 h compared to controls (untreated MEFs) (q-value < 0.05) after normalization with Z-score. Hierarchical clustering was constructed with Euclidean distance using R software. N = 3 biological replicas (3 independent experiments). (**b**–**c**) Enrichment analysis summarizing the most representative up-regulated pathways in TGFß1_up^genes^ (**b**) and TNFα_up^genes^ (**c**) using Metascape (http://metascape.org/gp/index.html#/main/step1).

First, we assessed the expression of *Col1a1*, *Acta2*, *Fn1*, *Mmp3*, *Il6* in MEFs after a stimulation with proinflammatory (TNFα) or profibrotic (TGFß1) cytokines at 6-h, 12-h, 18-h, and 24-h. We observed that the gene expression profile associated with “profibrotic effects” was more marked after a 24-h stimulation than after a shorter stimulation (upregulation of *Col1a1*, down regulation of *Mmp3* and *Il6*). Moreover, the distinction between TNFα- and TGFβ1-treated MEFs was more pronounced after a 24-h stimulation, especially with respect to *Mmp3*, *Fn1* and *Col1a1* (See Supplementary Fig. S1). For these reasons, we investigated the transcript expression of MEFs by RNA microarrays after a 24-h stimulation. With an absolute fold-change of 1.5, 1456 and 2215 distinct transcripts were significantly deregulated (q < 0.05) in MEFs treated by TNFα or TGFß1 compared to controls, respectively. Principal component analysis (PCA) showed that TNFα and TGFß1 induced two different gene expression profiles (See Supplementary Fig. S2). Based on the hierarchical clustering, we then focused analyses on clusters of interest of Differentially Expressed Genes (DEGs): (i) a cluster named TGFß1_up^g^ of 412 DEGs preferentially overexpressed in MEFs treated by TGFß1, and (ii) a cluster named TNFα_up^g^ of 242 DEGs preferentially overexpressed in MEFs treated by TNFα (See Fig. 1a). In these clusters of interest, a clustering enrichment analysis was performed to take account of redundancies between GO, KEGG and REACTOME terms. The analysis on TGFß1_up^g^ showed an enrichment in terms associated to the ECM organization (i.e., GO:0048729: tissue morphogenesis; R-MMU-1474244: extracellular matrix organization), while the analysis on TNFα_up^g^ showed an enrichment in terms associated to chemoattraction signaling (i.e., GO:0050900: leukocyte migration), immune response (i.e., GO:0050778: positive regulation of immune response), and inflammation response (i.e., GO:0006954: inflammation response) (See Fig. 1b–c; Supplementary Fig. S3; Table 1).

**Figure 2:**
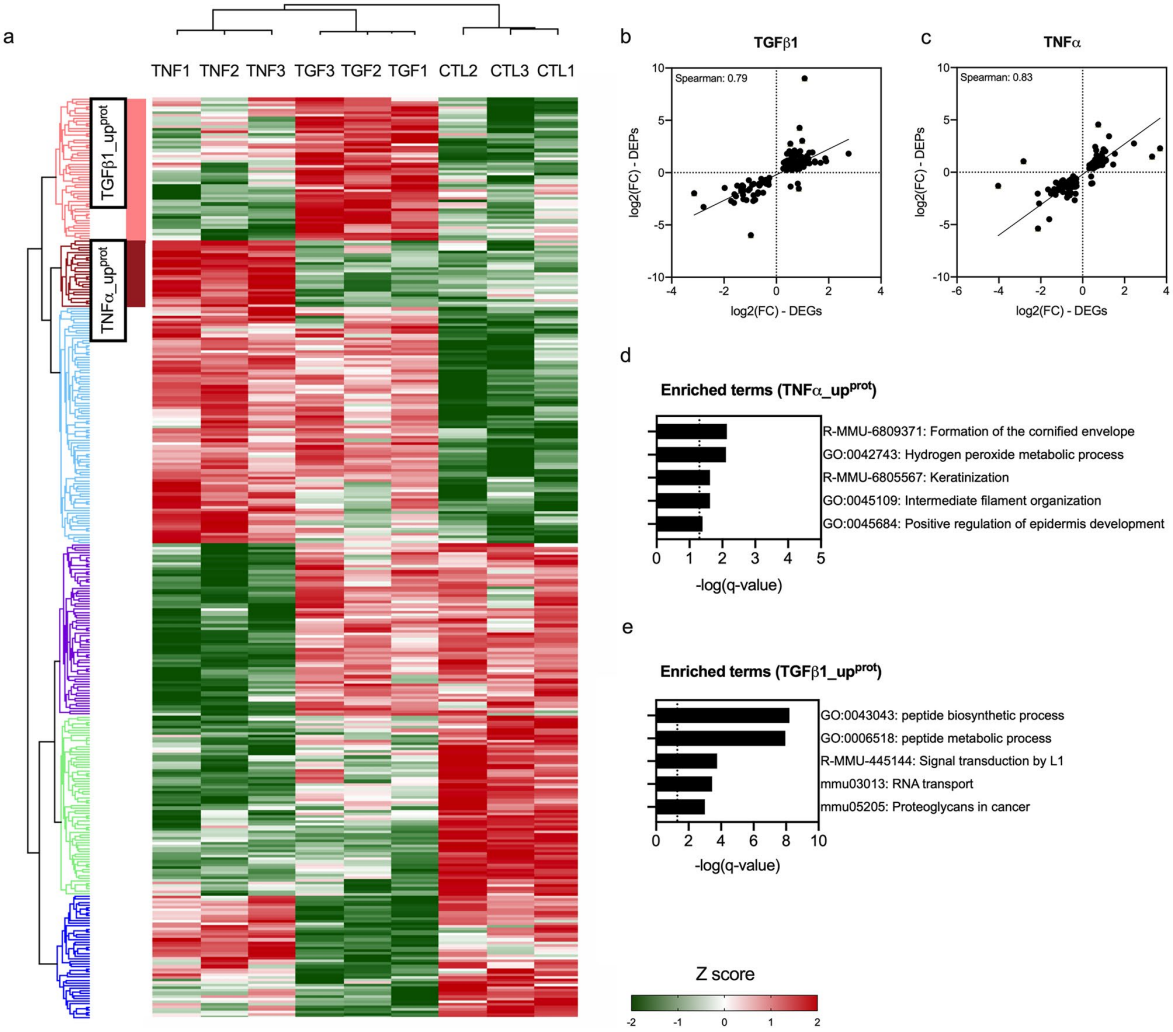
Proteome analysis of control, TGFß1- and TNFα-treated MEFs using LC–MS/MS. (**a**) Heatmap summarizing DEPs in TGFß1- (5 ng/ml) or TNFα- (10 ng/ml) treated MEFs for 72 h compared to untreated MEFs (controls; q-value < 0.05) after normalization with Z-score. Hierarchical clustering was constructed with Euclidean distance using Perseus software (https://maxquant.net/perseus). (**b**–**c**) Spearman correlation of DEGs expressed as log2(FC) assessed by RNA microarrays and DEPs as log2(FC) assessed by LC–MS/MS in TGFß1 (**b**; n = 150) and TNFα- treated (**c**; n = 110) MEFs compared to control MEFs. (**d**–**e**) Enrichment analysis summarizing the most representative up-regulated pathways in TGFß1_up^prot^ and in TNFα_up^prot^ (q-value < 0.05) using Metascape (http://metascape.org/gp/index.html#/main/step1). N = 3 biological replicas (3 independent experiments).

We next investigated the proteome using LC–MS/MS after a 72-h stimulation. Among the expressed proteins, 289 and 243 were significantly deregulated in MEFs (DEPs, Differentially Expressed Proteins) treated by TNFα or TGFß1 compared to controls, respectively (See Fig. 2a; Supplementary Fig. S4a–b). The Spearman correlation coefficients between the transcriptomic and proteomic expression were high (r = 0.79–0.83) (See Fig. 2b–c). PCA showed that these two cytokines induced two distinct protein expression profiles (See Supplementary Fig. S4c–e). As for RNA microarrays, we focused on: a cluster named TGFß1_up^prot^ of 57 DEPs preferentially over-expressed in MEFs treated by TGFß1 and a cluster named TNFα_up^prot^ of 27 DEPs preferentially over-expressed in MEFs treated by TNFα*.* MEFs treated by TGFß1 were enriched in terms associated with TGFß1 signaling (i.e., R-MMU-445144: signal transduction by L1) and ECM-modeling (i.e., mmu05205: proteoglycan in cancer), while MEFs treated by TNFα were enriched in pathways related to acute phase response (i.e., GO:0042743: hydrogen peroxide metabolic process) (See Fig. 2d–e; Supplementary Table S2).

### Selection of candidate markers related to the activation of pathways related to proinflammatory and profibrotic fibroblast polarizations

After confirming that the fibroblasts treated by TNFα or TGFß1 were representative of proinflammatory or profibrotic polarizations, we designed a gene signature to screen the activation of proinflammatory and profibrotic pathways. As LC–MS/MS did not allow the identification of smaller molecules such as cytokines, which are important mediators in the induction of these profiles, we focused on the process of selecting candidate genes based on transcriptome data, while including in our selection process the proteins identified by LC–MS/MS except for the smaller molecules.

**Figure 3:**
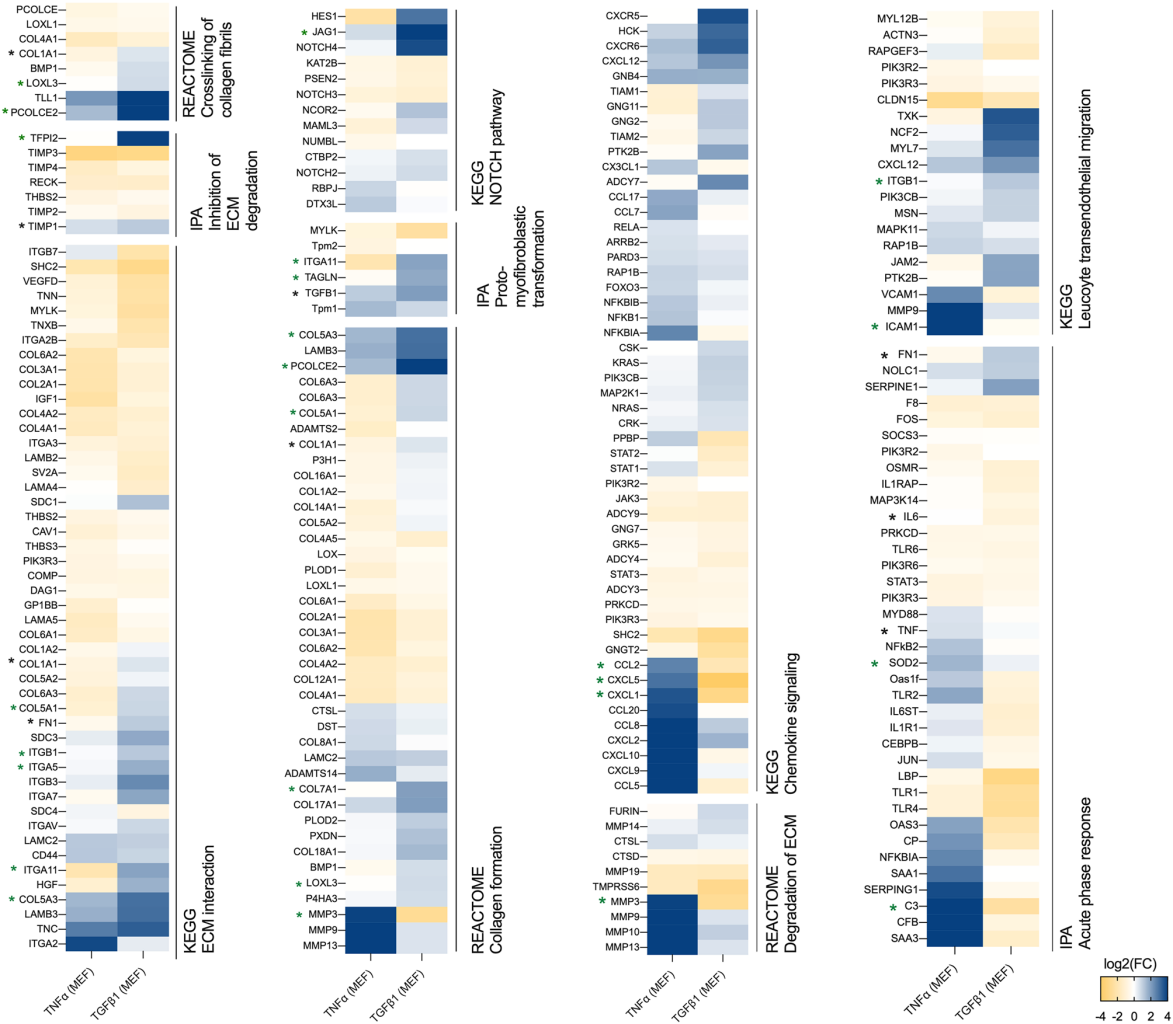
Gene expression of pathways related to the inflammatory response and to ECM organization in TGFß1- and TNFα-treated MEFs. Gene expression of pathways in TGFß1- and TNFα-treated MEFs expressed as log2 (Fold change vs. controls) assessed by RNA microarrays. Gene lists come from molecular signatures database (MSigDB) (http://software.broadinstitute.org/gsea/msigdb/index.jsp) and Ingenuity Pathway Analysis (Qiagen, Inc) (https://digitalinsights.qiagen.com). Only significant genes are presented in heatmaps (q-value < 0.05) generated using Prism (https://www.graphpad.com/scientific-software/prism/). Candidate genes and classical genes are respectively marked with green or black mark.

**Figure 4:**
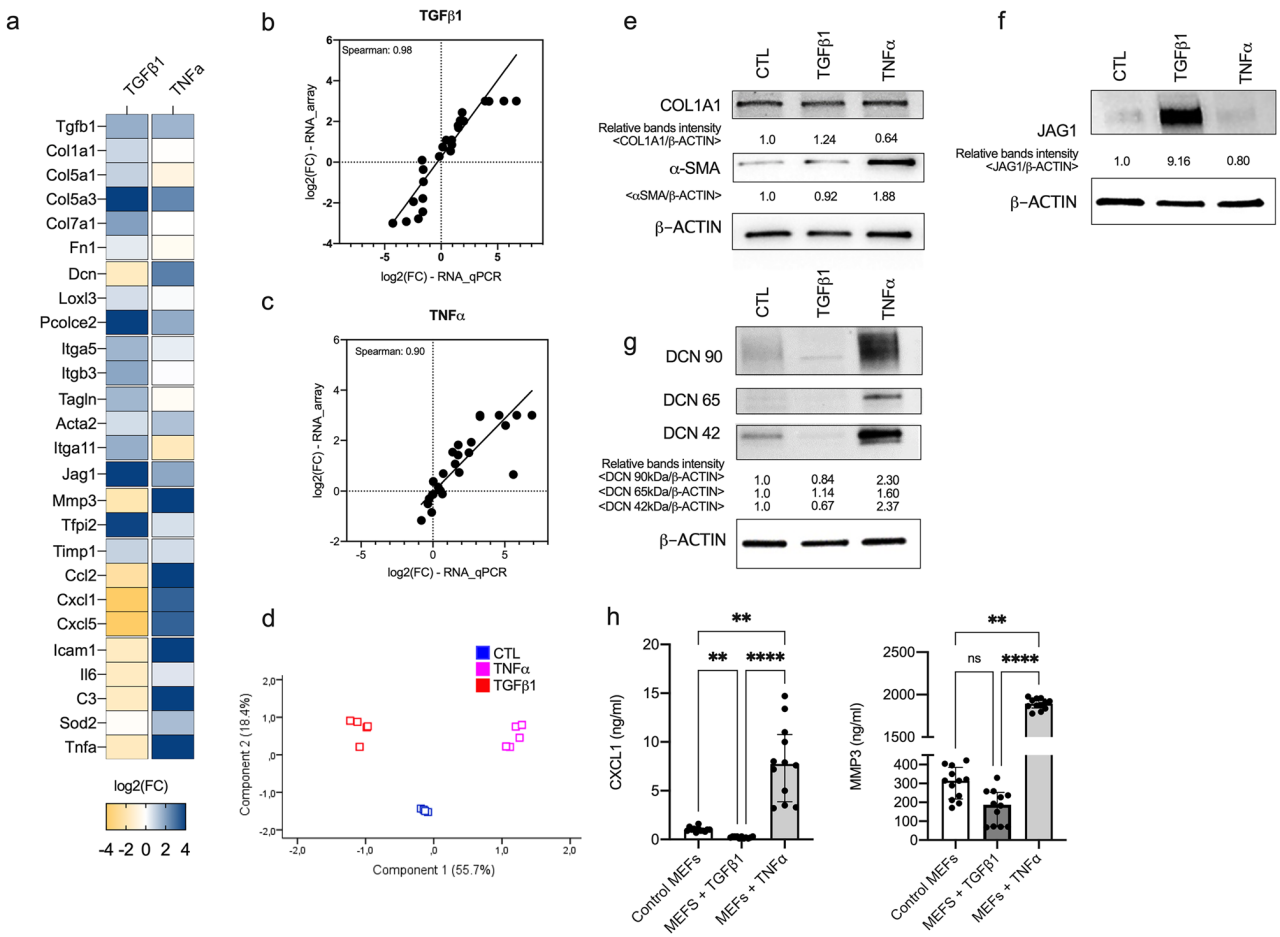
Gene signature expression. (**a**) Genes of gene set differentially expressed in TNFα-treated, TGFß1-treated MEFs compared to control MEFs assessed by RT-qPCR. Fold changes are log2-transformed and expressed as a double gradient colormap. Log2 values of fold changes are censored at − 4 or 4 for better viewing. (**b**–**c**) Spearman correlations of genes expressed as log2(FC) assessed by RNA microarray (at 24 h) and by RT-qPCR (at 24 h) in TGFß1- (**b**) and TNFα-treated (**c**) MEFs compared to control MEFs. (**d**) Principal component analysis of MEFs (controls, treated by TGFß1 or TNFα) assessed by RT-qPCR. (**e**–**g**) Western Blot analysis of level of COL1A1 (**e**), αSMA (**e**), JAG1 (**f**), and DCN (**g**) in control, TGFß1- and TNFα-treated MEFs. Intensities of specific bands was measured by densitometry. ß-ACTIN was used for normalization of data. Experiment is representative of n = 3 independent experiments. Full unedited Western Blot are provided in supplementary Figs. S8–S10. (**h**) Quantification of CXCL1 (ng/ml) and MMP3 (ng/ml) in the supernatant sample (n = 12/group; 3 independent experiments). Numbers are expressed as median with IQR.

We selected several pathways involved in the acute phase response (GO:0006954) and in the organization of ECM (R-MMU-1474244) with a particularly focus on the crosslinking of collagen fibrils (mmu2243919), collagen formation (R-MMU-1474290), ECM-receptor interaction (R-MMU-04512), acute phase response signaling (IPA), inhibition of Matrix Metalloproteases (IPA), proto-myofibroblastic transformation (IPA), degradation of ECM (R-MMU-1474228.1), chemokine signaling (mmu04062) and leukocyte transendothelial migration (mmu04670) (See Fig. 3; Supplementary Fig. S5). We applied the following criteria to select candidate genes: significance of q-value (vs. untreated MEFs), pathways membership (representativeness of meaningful biological processes), importance of the fold change (vs. untreated MEFs), distinctiveness of the 2 prototypic polarizations (i.e., up-regulated in one and down-regulated in the other), and if applicable the identification of the corresponding protein in the LC–MS/MS approach. The conventional markers (*Acta2*, *Fn1*, *Col1a1*, *Tgfß1*, *IL6, Timp1*) were systematically included. The full gene set was composed of: *C3*, *Ccl2*, *Cxcl1*, *Cxcl5*, *Icam1* (chemoattraction); *Dcn*, *Il6*, *Tnfα, Sod2* (acute phase response signaling pathway)*; Mmp3 (*ECM degradation); *Tfpi2*, *Timp1 (*regulation of ECM degradation)*; Col1a1*, *Col5a1*, *Col5a3*, *Col7a1* (collagens); *Fn1, Jag1*, *Loxl3*, *Pcolce2, TGFß1, Dcn* (ECM organization)*; Itga5*, *Itgb3* (ECM binding); *Acta2*, *Itga11*, *Tagln* (myofibroblastic transformation) (See Fig. 4a). The Spearman coefficients between the gene set expression assessed by RNA microarray and by RT-qPCR were high (range from 0.90 to 0.98) suggesting a very good correlation between the two methods. PCA showed that the gene signature assessed by RT-qPCR was able to separate the two polarizations of fibroblasts as accurately as RNA microarray did (See Fig. 4b–d; Supplementary Figs. S6–S7). We further analyzed the protein expression of some of these candidate markers either by ELISA assays in supernatant samples (MMP3, CXCL1) or by Western blot (COL1A1, α-SMA, JAG1, DCN). The results were consistent with the gene expression (See Fig. 4e–h; Supplementary Figs. S8–S10).

### Gene signature application in an experimental murine model of inflammation-mediated fibrosis

**Figure 5:**
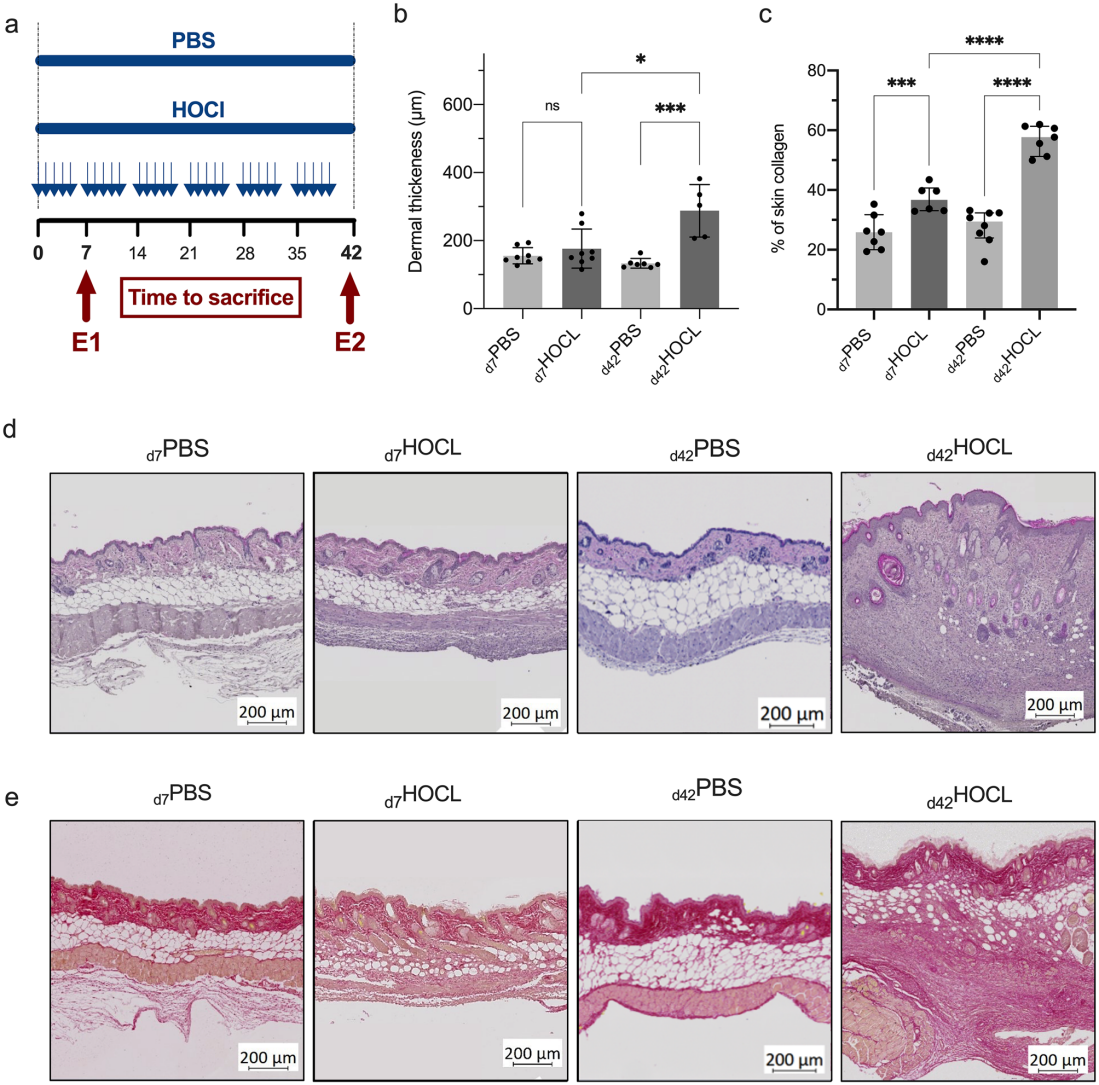
Inflammation-mediated fibrosis model. (**a**) Experimental procedure and sample collection times. Two independent experiments: E1 at the inflammatory stage (early stage); E2 at the fibrotic stage (late stage); (**b**) Dermal thickness estimated by measuring the distance between the dermoepidermal junction and the junction between the dermis and subcutaneous fat at 20X magnification using ImageJ morphometric software. Twenty random measurements were performed per section. (**c**) Collagen deposition in the skin evaluated using Picrosirius red staining expressed as % of the area occupied by collagen. (**d**) Representative sections of skin samples from PBS mice and HOCl mice under light microscopy after HE staining. (**e**) Representative sections of skin samples from PBS mice and HOCl mice at each time point, observed by light microscopy after staining with Picrosirius red. Medians with IQR are shown.

Repeated intradermal HOCl injections induced dermal inflammation and fibrosis in a time-dependent manner based on the oxidative stress theory^18^ (See Fig. 5a). Skin biopsy samples were performed at early stage (day 7—“inflammatory” stage, n = 84/group) and late stage (day 42—“fibrotic” stage, n = 84/group, 2 independent experiments).

First, we confirmed the proper induction of an inflammation-mediated fibrosis by investigating histological features. In PBS mice, we observed no significant increase of dermal thickness over time. In HOCl mice, the dermal thickness gradually increased from day 7 (median [IQR] = 156.0 µm [140.5; 230.0] vs. 148.5 µm [139.3; 180.8] in PBS, *p* = 0.67) to day 42 (304.0 µm [209.0; 358.5] vs. 130.0 µm [122.0; 134.0] in PBS, *p* < 0.001) (See Fig. 5b). Histology showed a strong collagen deposition in the skin of HOCl mice compared to PBS mice at day 7 (mean ± SD = 37.1% ± 4.4 vs. 25.9% ± 5.9, *p* = 0.004), increasing progressively over time in HOCl mice (at day 42: 56% ± 4.9 vs. 27.7 ± 5.9 in PBS) (See Fig. 5c). At day 42 in HOCl mice, we observed a loss of normal skin architecture in accordance with skin fibrosis at the late stage (See Fig. 5d–e). The percentage of leukocytes among all skin cells was higher in HOCl mice compared to PBS mice on day 7 (mean ± SD = 33.7% ± 9.5 vs 13.0% ± 4.7, respectively, *p* = 0.002), then gradually decreased at day 42 (14.3% ± 7.1 vs 8.3% ± 4.0, respectively, *p* = 0.14) in line with an early inflammatory stage (n = 4–5/group).

**Figure 6:**
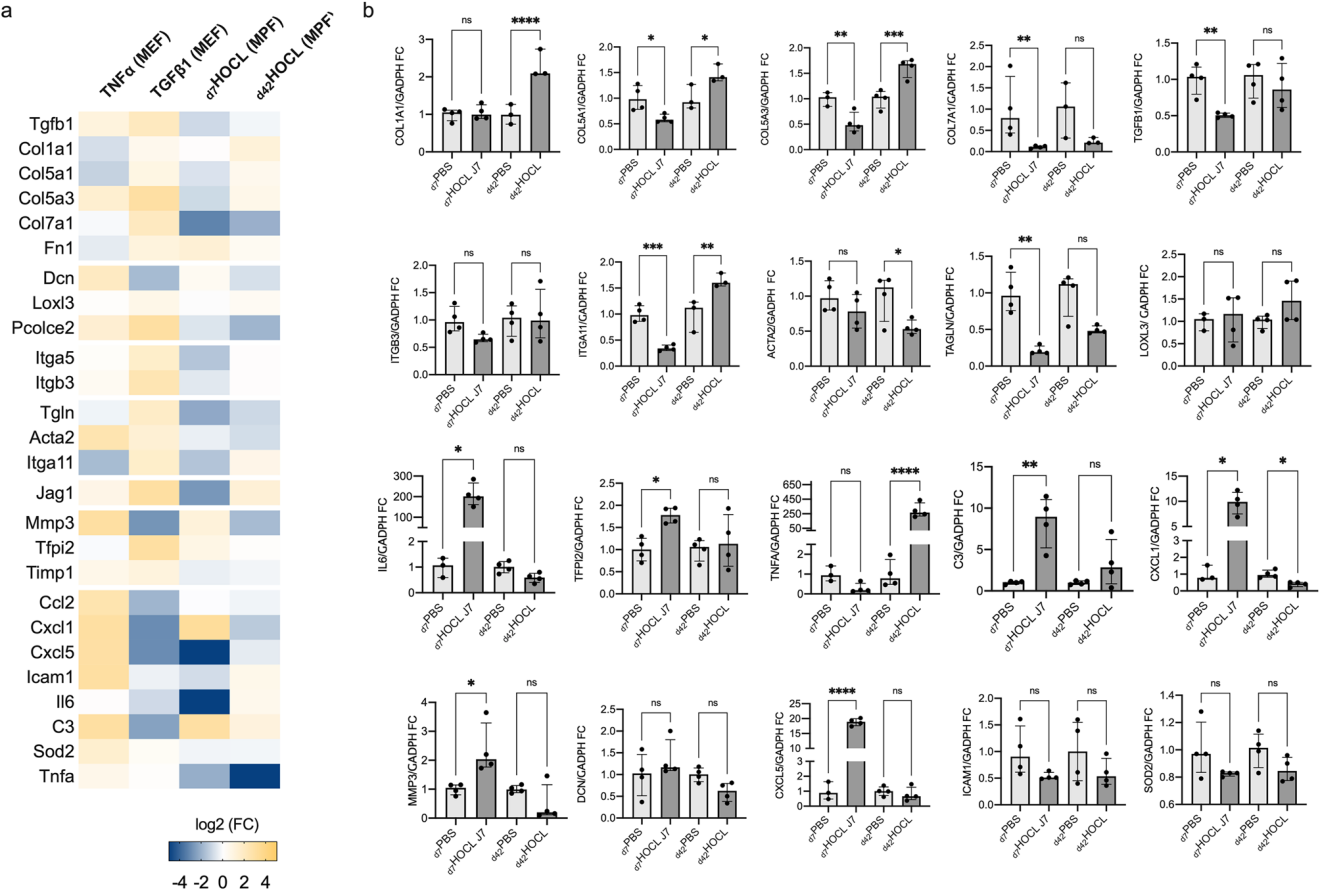
Gene signature expression assessed by RT-qPCR in HOCL mice and in PBS mice at day 7 and day 42. (**a**) Heatmap showing the transcript gene set expression assessed by RT-qPCR from (i) TNFα-treated MEFs (compared to control MEFs), (ii) TGFß1-treated MEFs (compared to control MEFs), (iii) d_7_HOCL MPFs (compared to d_7_PBS MPFs), and (iv) d_42_HOCL MPFs (compared to _d42_PBS MPFs). Fold changes are log2-transformed and expressed as a double gradient colormap. Log2 values of fold changes are censored at -5 or 5 for better viewing. The heatmap was generated using Prism (https://www.graphpad.com/scientific-software/prism/), (**b**) RT-qPCR results. Numbers are expressed as median fold change (IQR) compared to PBS at day 7 or day 42. *P*-value: *** ≤ 0.001, ** ≤ 0.01, * ≤ 0.05. ns: > 0.05.

We investigated the gene signature expression of dMPFs isolated from skin biopsy samples. At day 7, we observed a proinflammatory polarization of dMPFs in _d7_HOCL mice (n = 4, 1 experiment) compared to _d7_PBS mice (n = 4, 1 experiment) characterized by: (i) the over-expression of the genes related to chemoattraction, acute phase response signaling and ECM degradation; and (ii) the down-expression of the genes related to ECM biosynthesis and myofibroblast transformation. *Tnf*α was under-expressed in _d7_HOCL mice compared to _d7_PBS mice. At day 42, we observed a mixture of a proinflammatory and profibrotic polarizations of MPFs in _d42_HOCL mice compared to _d42_PBS mice characterized by: (i) few inflammatory features, remaining lower than those observed in _d7_HOCL mice, excepted a marked up-regulation of *Tnf*α expression (*p* < 0.001); (ii) the overexpression of genes related to ECM-biosynthesis and ECM-remodeling; and (iii) the down-expression of genes related to ECM-degradation (See Fig. 6, Supplementary Fig. S11).

## Discussion

Nowadays, transcript gene expression of fibroblast polarization is mostly based either on the expression analysis of a few genes with the bias of missing a significant effect due to inaccurate pre-selected targets, or on more in-depth analyzes such as RNA microarrays or RNA sequencing which take time and resources. This study provides an original process to propose a convenient and feasible gene signature to analyze fibroblast polarization.

First, we selected candidate genes by following an original multi-stage pathological process. To induce the profibrotic fibroblast in vitro, we treated MEFs with TGFβ1 conventionally used as a positive control for fibrosis^4,20^. To induce the proinflammatory profile in vitro, we treated MEFs with TNFα which is produced by macrophages during the inflammatory phase of wound healing^21^. The transcript gene expression and the gene ontology analysis associated with the proinflammatory and profibrotic MEFs induced in vitro were consistent with previous data on profibrotic and proinflammatory polarizations of fibroblasts^4,10,22^. We selected the most representative pathways, chose representative genes of each pathway and combined them to illustrate the proinflammatory and profibrotic polarizations. Second, we assessed the gene signature on dMPF isolated from an experimental murine model of inflammation-mediated fibrosis in a time-dependent manner. Maria et al. showed that daily injections of HOCl triggered inflammation and led to cellular polymorphous infiltrates (inflammatory stage), following by a progressive development of established fibrosis, less inflammatory, and made of disorganized collagen fibers destructuring all skin layers^19^. At the inflammatory stage, we observed a proinflammatory polarization of the dMPF, while we observed a mixture of proinflammatory and profibrotic polarization at the fibrotic stage in consistence with the histological findings and the pathogenesis of fibrosis in this model^16–19^.

The gene signature was composed both of conventionally-used genes (sometimes with a low power of discrimination such as *Acta2)* and innovative genes selected based on the importance of the fold change, the membership in meaningful biological processes and on the differential deregulation between the proinflammatory or profibrotic polarizations.

Several studies have shown that the ECM overproduction is not limited to *Acta2* + fibroblasts and that *Acta2* should not be used as a single marker of myofibroblastic transformation^9,10,13,14^. That is the reason why we selected *Tagln*^9,12,14^ and *Itga11* to increase the detection of myofibroblastic transformation^23–25^. In our study, we observed an increased expression of *Acta2* at the level of gene and protein expressions in the two prototypical polarizations, which highlighted that an interpretation based on a few markers could lead to a misinterpretation of fibroblast polarization. It was therefore important to add other features to assess the profibrotic polarization, such as matrix crosslinking, matrix degradation, and matrix microenvironment. Matrix crosslinking regulate matrix stiffness^26–28^. Lysyl oxidases (LOXs) and lysyl oxidase‐like 1‐4 (LOXL 1‐4) are a group of enzymes that catalyzes cross-linking of collagens, thereby rendering these matrix proteins unable to be degraded. They have been shown to be positively correlated with fibrosis in numerous organs including kidney, heart, liver, and lung^10,11,27,29,30^. Additionally, recent studies have shown that LOX(L) inhibition can lead to reduction in activated fibroblasts, in collagen content and in crosslink formation, making *Loxl3* a marker of interest^31,32^. Degradation and inhibition of the degradation of the ECM should be also considered to explore fibroblast polarization. We selected *Mmp3* and 2 inhibitors of MMPs to assess this balance. A potent inhibitor of metalloproteases is *Tfpi2* which cleaves the serine proteases involved in the cleavage of biologically active pro-MMPs. *Timp1* is one of the other major inhibitors of metalloproteases overexpressed in fibrotic diseases^33^. The ECM is a very complex three-dimensional structure that is not only limited to a mechanical support role but whose elements are also involved in various signaling pathways and cellular processes, including inflammation, proliferation, apoptosis, and angiogenesis. Among these elements, proteoglycans critically provide structure to the ECM, but also regulate many signaling pathways, such as growth factors, cytokines, chemokines, and matrix-cell contacts^6,26,34^. Decorin is a crucial proteoglycan, also called “the guardian from the matrix”, that “decorates” collagen fibrils and regulates the cell cycle by trapping TGFß^34,35^. Decorin has also the ability to act as a pan-receptor inhibitor of tyrosin kinases, including epidermal growth factor receptor, vascular endothelial growth factor receptor 2, mesenchymal-epithelial transition factor receptor; thus restraining angiogenesis^26,36^. The selection of decorin brings new insights in the assessment of fibroblast polarization.

In a work on primary murine fibroblasts isolated from healthy lung or experimental pulmonary fibrosis, Akamatsu et al. showed that: (i) “inflammatory” fibroblasts significantly overexpressed genes associated with collagens and chemokines compared to quiescent fibroblasts; (ii) myofibroblasts overexpressed genes associated with ECM- synthesis and crosslinking, and underexpressed genes associated with chemokines compared to quiescent fibroblasts^10^. We selected several relevant inflammatory markers which have already been reported at the inflammatory phase of wound healing and of bleomycin-induced alveolitis to assess the inflammatory features associated with proinflammatory polarization^10,21^.

Previous studies on murine fibroblasts showed *Col1a1* and *Col5a1* were overexpressed both in inflammatory fibroblasts and in myofibroblasts^12,14^, which is consistent with our results. Some collagens seem to be preferentially expressed in myofibroblasts (*Col7a1*) or inflammatory fibroblasts (*Col5a3*, *Col14a1*)^14^. We have therefore selected several types of collagens to better describe this heterogeneity. In a single-cell analysis performed at day 21 in a mouse model of pulmonary fibrosis induced by bleomycin, Xie et al. identified two subclusters among *Col1a1* + fibroblasts: *Col13a1* matrix fibroblasts with profibrotic features and *Col14a1* matrix fibroblasts with proinflammatory features such as up-regulation of *Col5a3*, *Cxcl12*, *Mmp3* and *Dcn*^14^*.* These results are very consistent with features of proinflammatory and profibrotic polarizations delineated by our gene signature, while the gene expression of *Col14a1* and *Col13a1* were not significant in this study. Overall, our results suggest that this gene signature could be meaningful, and the small size of the gene list makes it a convenient way to precisely assess fibroblast polarizations, for example to screen drugs. An analysis based solely on the conventionally-used genes would not have illustrated neither the importance of the proinflammatory features of fibroblasts at d7 (*Il6*, but also chemoattraction factors), nor the profibrotic multidimensional features of fibroblasts at d42 (activating of *Notch* pathway, proto-myofibroblastic transformation, ECM biosynthesis, negative regulation of ECM degradation). Our work has some limitations. We developed the gene signature from gene expression of MEFs. Our application on dMPFs confirms its applicability, but further work will be necessary to assess its interest in other organs and in tumoral microenvironment. Indeed, we did not assess the gene signature to explore cancer associated fibroblast (CAF) polarization. However, single cell RNA sequencing of several cancer types demonstrated the presence of transcriptionally distinct CAF population^37,38^. Across various cancer types, myofibroblastic (αSMA-high) CAFs are associated with a matrix-producing contractile phenotype, whereas inflammatory (αSMA-low) CAFs are generally specialized in inflammatory cytokine and chemokine secretion^37–40^. Given the similarities between, on the one hand, the profibrotic fibroblast with the myofibroblastic CAF, and one the other hand, the proinflammatory fibroblast with the inflammatory CAF, it could be relevant to explore the CAF polarization in the tumoral microenvironment using the gene signature.

We provide here an original detailed multi-stage process to develop a gene signature to analyze fibroblast polarization, including its confirmation in an experimental model of inflammatory fibrosis. This gene signature will allow to be more precise than the usual single markers currently used and more feasible and convenient that the complex full transcriptomic approach.

## Methods

### Mouse embryonic fibroblasts

#### Cells

MEFs were purchased from American Type Culture Collection (Balb/c/3T3 clone A31, ATCC, MD, USA) and were used between 8 and 15th passages.

#### Reagents and cell cultures

MEFs were cultured in DMEM Glutamax (Dulbecco’s Modified Eagle Medium) supplemented with fetal calf serum (FCS), penicillin, and streptomycin (all from Thermofisher). MEFs (10^5^ cells) alone or stimulated were seeded in 12-well plates for 24 or 72 h. For MEFs stimulation, cells were incubated either with 5 ng/ml of recombinant TGFβ1 (R&D Systems Lille, France) or with 10 ng/ml of recombinant TNFα (R&D Systems Lille, France).

### Experimental murine model of inflammation-mediated fibrosis

#### Animals

Six-week-old female BALB/c mice (Janvier Labs, France) were used in all experiments. Animals were housed in a specific pathogen-free facility, within autoclaved ventilated cages with sterile food and water ad libitum, under constant room temperature and with 12-h day–night cycles. This study was carried out in accordance with the local and national guidelines (directive #68/609 CEE). The protocol was approved by the Regional Ethics Committee on Animal Experimentation.

#### Experimental procedure

Experimental model was induced by daily intradermal injections of 300 μl of an HOCl-generating solution into the shaved backs of mice, using a 27-gauge needle and a 1-ml syringe, as previously described. The HOCl-generating solution was extemporaneously prepared by adding NaClO solution (9.6% as active chlorine) to a 100 mM KH2PO4 solution (pH 6.2). The NaClO amount was determined by measuring the optical density (OD) of the solution at 280 nm, and then adjusted to obtain an OD between 0.7 and 0.9. Control mice received injections of 300 μl of sterilized phosphate-buffered saline (PBS).

#### Sample collection

Mice were sacrificed by cervical dislocation under deep CO2 anesthesia at either day 7 (inflammatory stage) or day 42 (fibrotic stage) after the first injection. Skin samples were collected at the time of euthanasia near the injection site.

#### Dermal mouse primary fibroblasts culture

To isolate dMPFs, the skin was harvested, minced into small pieces, and incubated in PBS containing dispase II (5 mg/ml) overnight at 4 °C. After removing epidermis, the dermis was incubated in 1X PBS containing collagenase IV (4 mg/ml) for 1 h. Cells were pelleted by centrifugation, washed twice, and grown in DMEM containing 10% FBS. Cells were used at the second passage.

#### Histological evaluation

Skin samples embedded in paraffin were sliced into serial 4-μm sections. Dermal thickness at the injection site was assessed by performing a hematoxylin eosin (HE) staining and measuring the distance between the epidermal–dermal junction and the dermal–subcutaneous fat junction at a 20-fold magnification using the ImageJ software (U.S. National Institute of Health) (https://imagej.nih.gov/ij/index.html). Twenty random measurements per section were performed by two blinded investigators and averaged for each section.

#### Measurement of collagen deposition

Collagen deposition in the skin was evaluated using Picrosirius red staining. The color was prepared by a 5-min bath of hematoxylin diluted 1/3 in water (Bio Optical). A 60 min bath of Picrosirius Red, which specifically dyes collagen fibers, was then carried out, obtained by mixing Direct red 80 (Sigma Aldrich) diluted to 1% in saturated picric acid (Sigma Aldrich). The sections were mounted between slide and coverslips and acquired under an automated microscope as described above, then analyzed using the ImageJ software (U.S. National Institute of Health) (https://imagej.nih.gov/ij/index.html). A semi-quantitative analysis of the collagen deposition was performed using a method of Color deconvolution and the area occupied by collagen was quantified (expressed in %).

#### Leukocytes count in skin sample

After collection, the skin biopsies were digested with type II dispase (1 mg/mL to 1.88 units/mL) and type IV collagenase (0.5 mg/mL to 215 units/mL) for 2h30 at 37 °C. The suspension was then centrifuged at 300 g for 10 min. Cell counting was performed by flow cytometry (NaviosTM, Beckman Coulter) using calibrated beads (Flow-Count Fluorospheres, cat. # 7547053, Beckman Coulter). 50 μL of cell suspension were incubated with 2 μL of anti-murine Fc receptor antibody (Fc block, BD Pharmingen) for 5 min at 4 °C. They were then incubated with 5 μL of CD45-APC (Biolegend) for 20 min at 4 °C. The samples were then incubated with 2.5 µL of propidium iodide (Bioscience) for 5 min at 4 °C. Data were analyzed using Kaluza Analysis software (Beckman coulter, version 2.1) (https://www.beckman.fr/flow-cytometry/software/kaluza).

### Evaluation of polarizations of fibroblasts

#### Gene expression analysis using RNA microarray

##### Library preparation and data acquisition

Total RNA yield and quality were assessed on the Agilent 2100 bioanalyzer (Agilent Technologies. Massy, France). One color whole Mouse (074809_D_F_20171030 design) 60-mer oligonucleotides 8 × 60 k microarrays (Agilent Technologies) were used to analyze gene expression. cRNA labelling, hybridization and detection were carried out according to supplier’s instructions (Agilent Technologies). For each microarray, Cyanine 3-labeled cRNA were synthesized with the low input QuickAmp labeling kit from 50 ng of total RNA. RNA Spike-In were added to all tubes and used as positive controls of labelling and amplification steps. The labelled cRNA were purified and 600 ng of each cRNA were then hybridized and washed following manufacturer’s instructions. Microarrays were scanned on an Agilent G2505C scanner and data extracted using Agilent Feature Extraction Software© (FE version 10.7.3.1). Microarray data have been submitted to the GEO database under the accession number GSE191223.

##### RNA microarray data analysis

We analyzed RNA microarray data using Linear Models for Microarray Analysis (LIMMA) package in R software (version R 3.6.0) (https://www.r-project.org) to identify genes that were expressed differentially between datasets. The threshold for statistical significance was set to q-value (Benjamini–Hochberg false discovery rate (FDR) at < 0.05. Normalization with Z-score was determined, and hierarchical clustering was constructed with Euclidean distance.

#### Protein identification using LC–MS/MS

##### Sample preparation and LC–MS/MS Orbitrap eFASP

Samples were prepared using a modified enhanced Filter Aided Sample Preparation (eFASP) in order to increase proteome coverage and sample recovery for quantitative proteomic experiments^41^. LC–MS/MS protein analysis was performed on an Orbitrap Q Exactive plus Mass Spectrometer hyphenated to a U3000 RSLC Microfluidic HPLC System (ThermoFisher Scientific). Detailed procedure is available in the Supplementary file 1.

##### Quantification MaxQuant

Analysis of raw file from LC–MS/MS eFASP digestion data was performed using MaxQuant v1.5.3.30. and Andromeda search engine was used for database searching against the UniProtKB/Swiss-Prot Mouse database (Mus musculus, January 2018, Sequences: 91.097). MaxQuant also contains common contaminates proteins identified in proteomics analysis. MaxQuant analysis included an initial search with a precursor mass tolerance of 20 ppm, a main search precursor mass tolerance of 6 ppm and a fragment mass tolerance of 20 ppm, respectively. Trypsin was selected as an enzyme and 3 missed cleavages are included. Together with variable modifications such as methionine and proline oxidation, deamidation on asparagine or glutamine and serine, threonine phosphorylation, glutamate to pyroglutamate conversion and with the fixed modification carbamidomethyl cysteine. The minimal peptide length was set to six amino acids and the maximum number of missed cleavages to three. The match-between-runs function was used (match time window = 2 min, alignment time window = 20). The FDR was set to 0.01 for both peptide and protein identifications. The proteins identified by the same sets of peptides were grouped and reported as one protein group. Proteomic data have been submitted to the PRIDE database under the accession number PXD034078.

##### Bioinformatic treatment by Perseus

The statistics were calculated using Perseus software (version 1.60.2. Max Planck Institute of Biochemistry, Martinsried, Germany) (https://maxquant.net/perseus/)^42^. The MaxQuant data were filtered for reverse proteins identifications (false positives), contaminants, and proteins “only identified by site”. The label free intensities were transformed in log2 then the data were filtered with x valid values in at least one group. The missing data were replaced from normal distribution (width 0.3. down shift 1.8). Significant proteins were determined using a two-sample analysis t-test and multiple sample test with Benjamini–Hochberg FDR at 0.05. Normalization with Z-score was determined, and hierarchical clustering was constructed with Euclidean distance.

#### real-time quantitative PCR (RT-qPCR)

Total RNA was extracted from fibroblasts (MEFs or MPFs) by using a Nucleospin RNA kit (Macherey–Nagel, Hoerdt, France). Detailed procedure and sequence of each primer are available in the Supplementary file 1. All samples were amplified in duplicate. Analysis of relative gene expression data was performed using the 2^−ΔΔCt^ methods, where ΔCt was the difference in crossing points between housekeeping gene (*Gapdh*) and gene tested.

#### Western blotting

Cells were lysed using RIPA Buffer (Tris 50 mM; NACl 150 mM; SDS 0.1%; Na Deoxycholate 0.5%; Triton X100 1%) containing EDTA-free protease inhibitors (Roche, Missisauga, Canada), 1 mM PMSF, and 1 mM sodium fluoride. Lysates were clarified at 12000 g for 10 min and supernatants were normalized for protein concentration using the Bio-Rad DC Protein Assay (Bio-Rad, Hercules, CA). 25 μg proteins were separated on a 4–12% SDS-PAGE then transferred to nitrocellulose membrane. After blocking for 1 h in 10% BSA in TBS Tween buffer, membranes were probed with the following antibodies specific for collagen I (1:1000, #MA1-26771Thermofischer scientific), α-SMA (1:5000, ab7817, Abcam), decorin (1:400, #AF1060, R&D systems), jagged1 (1:1000, #70109, Cell Signaling Technology Inc.). Horseradish peroxidase-conjugated secondary antibodies were used at 1:2000 for 1 h then detection was carried out by enhanced chemiluminescence. Optical density of target bands will be determined by using the ImageJ software (U.S. National Institute of Health) (https://imagej.nih.gov/ij/index.html).

#### ELISA assays

CXCL1 and MMP3 protein levels in supernatant samples were assessed in duplicate using ELISA assays (Mouse Total MMP-3 Duoset Elisa, cat. #DY548; Mouse CXCL1 Duoset Elisa, cat. #DY453; R&D systems), at appropriate dilutions. All experiments were conducted according to the manufacturer’s protocols.

#### Ontology annotation and canonical pathway enrichment

To analyze Gene Oncology (GO), REACTOM, and KEGG pathways, we subjected differentially expressed genes (DEGs) (or proteins, DEPs) with cut-off q-value at < 0.05 to the online tool Metascape (http://metascape.org/gp/index.html#/main/step1)^43^. All significant enriched terms (*p*-value < 0.05) were hierarchically clustering into a tree based on Kappa-statistical similarities (threshold > 0.3) among their gene membership. The clusters identified have been named according to the membership. Among the terms within each cluster, some of them (q-value < 0.05) were selected for charting. Molecular signatures database (MSigDB) (http://software.broadinstitute.org/gsea/msigdb/index.jsp) and Ingenuity Pathway Analysis (Qiagen, Inc) (https://digitalinsights.qiagen.com) were used to identify candidate genes in selected gene set.

### Statistical analysis

Values were reported as median ± interquartile range or mean ± SD according to the normality of distribution checked graphically and using the Shapiro–Wilk test. Comparisons of groups were performed using the analysis of variance or the Kruskal–Wallis test. In the case of significance results, the pairwise comparisons were performed. Comparisons were performed using Student t test for gaussian continuous variables and using Mann Whitney U test for non-gaussian continuous variables. Correlation was performed using Spearman correlation. All statistical tests were performed at 2-tailed α level of 0.05. All statistical analyses were performed on Prism (version 9.0.2 GraphPad Software) (https://www.graphpad.com/scientific-software/prism/). Venn’s diagrams were created using Venny^2.1^ (BioinfoGP—Csic) (https://bioinfogp.cnb.csic.es/tools/venny).

### Ethics approval and consent to participate

This study was carried out in accordance with the local and national guidelines (directive #68/609 CEE). The protocol was approved by the Regional Ethics Committee on Animal Experimentation CEEA 75 (Comité d’éthique sur l’expérimentation animale) under the reference number: APAFIS#19603-2020061914271271 v6.

### Additional statement

The study was carried out in compliance with the ARRIVE guidelines.

## Supplementary Information

Supplementary Information 1.

Supplementary Information 2.

Supplementary Information 3.

## Data Availability

Transcriptomics datasets related to this article can be found at https://www.ncbi.nlm.nih.gov/geo/query/acc.cgi?acc=GSE191223, an open-source online data repository hosted at NCBI/GEO. GEO accession number: GSE191223. Proteomic data have been submitted to the PRIDE database under the accession number PXD034078.

## Abbreviations

ECM: Extracellular matrix
DEGs: Differentially expressed genes
DEPs: Differentially expressed proteins
FC: Fold change
FDR: False discovery rate
GO terms: Gene Ontology terms
HOCl: Hypochloric acid
LC–MS/MS: Liquid Chromatography with tandem mass spectrometry
MEF: Mouse embryonic fibroblast
dMPF: Dermal mouse primary fibroblast
PBS: Phosphate-buffered saline
