# Supplementary material for: Simple gene signature to assess murine fibroblast polarization
Source: Sci Rep. 2022 Jul 11;12:11748. doi: 10.1038/s41598-022-15640-6 (PMC9273630; doi:10.1038/s41598-022-15640-6)

SUPPLEMENTARY FILE

Title:

Simple gene signature to assess the murine fibroblast polarization

Authors:

Emmanuel Ledoult, Manel Jendoubi, Aurore Collet, Thomas Guerrier, Alexis Largy, Silvia Speca, Solange Vivier, Fabrice Bray, Martin Figeac, Eric Hachulla, Myriam Labalette, Frédéric Leprêtre, Shéhérazade Sebda, Sébastien Sanges, Christian Rolando, Vincent Sobanski, Sylvain Dubucquoi, David Launay.

SUPPLEMENTARY METHODS

Gene Expression Analysis Using RNA Microarray

Library preparation and data acquisition

Total RNA yield and quality were assessed on the Agilent 2100 bioanalyzer (Agilent Technologies. Massy, France). One color whole Mouse (074809_D_F_20171030 design) 60-mer oligonucleotides 8x60k microarrays (Agilent Technologies) were used to analyze gene expression. cRNA labelling, hybridization and detection were carried out according to supplier's instructions (Agilent Technologies). For each microarray, Cyanine 3-labeled cRNA were synthesized with the low input QuickAmp labeling kit from 50 ng of total RNA. RNA Spike-In were added to all tubes and used as positive controls of labelling and amplification steps. The labelled cRNA were purified and 600 ng of each cRNA were then hybridized and washed following manufacturer's instructions. Microarrays were scanned on an Agilent G2505C scanner and data extracted using Agilent Feature Extraction Software© (FE version 10.7.3.1). Microarray data have been submitted to the GEO database under the accession number GSE191223.

Real-Time Quantitative PCR (RT-qPCR)

Total RNA was extracted from fibroblasts (MEFs or MPFs) by using a Nucleospin RNA kit (Macherey-Nagel, Hoerdt, France). One microgram of total RNA was used to obtain single-stranded cDNA by using a specific Reverse Transcription Kit (Thermo Fisher Scientific) according to the manufacturer's protocol. Quantitative RT-PCR was performed by using LightCycler FastStart DNA Master SYBR Green I (Thermo Fisher Scientific), according to the manufacturer's protocol. Primers were made from available gene sequences (UCSC, version mm9) using Oligo7.60 (Molecular Biology Insights, Inc.). All samples were amplified in duplicate. Melting-curve analysis was performed to assess the specificity of PCR products. Analysis of relative gene expression data was performed using the $2^{-\Delta\Delta Ct}$ methods, where ΔCt was the difference in crossing points between housekeeping gene (*Gapdh*) and gene tested.

Primers used in RTqPCR experiments

	**Forward (5'-3')**	**Reverse (5'-3')**
Acta2	CCTGACGGGCAGGTGATC	ATGAAAGATGGCTGGAAGAGAGTCT
C3	CAACGCAAGTTCATCAGCC	GCTGGTGTTGGGCTTTTC
Ccl2	CTGCTGCTACTCATTCACC	GGTGACAAAAACTACAGCTTC
Col1a1	GAGTACTGGATCGACCCTAACCAA	ACACAGGTCTGACCTGTCTCCAT
Col5a1	CACAGGCAGCTATGATAAGG	CTTTCTTGGTAGCACAGCC
Col5a3	CAGAACTCGGTGGCATGGC	CTTCCGGACCCGACAGCC
Col7a1	CATCCCTTTGTCTATGGTGGC	CAGCACCTGTTTTCTGGCTG
Cxcl1	GCGCCTATCGCCAATGAG	GAGTGTGGCTATGACTTCGG
Cxcl5	GGGCAGTGACAAAAAGAAAGC	CAGCCCTTTCTTCTTATCTTCAC
Dcn	AACAGCATCACCGTTATGG	GACGACCTGGATATACTTATGC
Fn1	GATGCTCCCACTAACCTCCA	CGGTCAGTCGGTATCCTGTT
Gadph	ATGGGAAGCTTGTCATCAACG	GGCAGTGATGGCATGGACTG
Icam1	GTCAAACAGGAGATGAATGG	GAGAGTGGTACAGTACTGTC
Itga5	CAGCCGTGCAGTGGACCAAG	GCCGAGCTTGTAGAGGACG
Itga11	CAAGAAGACTGGCAGGTC	CTTTTAAAAAAACCGAGCTTCCAC
Itgb3	GCTCATTGGCCTTGCTACTC	GGTTGTTTGCTGTGTCCCAC
Il6	CAGAATTGCCATTGCACAAC	ACTGGCAAAAGGATGGTGAC
Jag1	CATCCGGGATGATGGGAAC	GGAACCAGGAAATCTGTTCTG
Loxl3	CCAAGAGGTATGAGTGCG	GTTGATAACGACCTGAAGAATGTAG

Mmp3	ACATGGAGACTTTGTCCCTTTTG	TTGGCTGAGTGGTAGAGTCCC
Pcolce2	CAGCATCTACAGGGAAGG	GTAATTGAGACCTCGTCTGAG
Sod2	GCCGTGTCTGTGGGAGTC	GCGGAATAAGGCCTGTTGTTC
Tgfß1	CCCGAAGCGGACTACTATGCT	GTTTTCTCATAGATGGCGTTGTTG
Tnfα	CCACCACGCTCTTCTGTCTA	GAGGCCATTTGGGAACTTCT
Tagln	GTAAGGATATGGCAGCAGTG	CCTGGGCTTTCTTCATAAAC
Timp1	GCCTAAGGAACGGAAATTTGC	GGAAACACTGTGCACACC
Tfpi2	GATGAAGGTCTGTGTTCTGC	CTTCTTCCAGCCTTTAACGC

Acta2: Actin, alpha 2, smooth muscle; *C3*: Complement component 3; *Ccl2*: C-C motif chemokine ligand 2; *Col1a1*: Collagen 1, alpha-1 chain; *Col5a1*: Collagen 5, alpha-1 chain; *Col5a3*: Collagen 5, alpha-3 chain; *Col7a1*: Collagen 7, alpha-1 chain; *Cxcl1*: C-X-C motif chemokine ligand 1; *Cxcl5*: C-X-C motif chemokine ligand 5; *Dcn*: Decorin; *Fn1*: Fibronectin 1; *Gadph*: Glyceraldehyde 3-phosphate dehydrogenase; *Icam1*: Intercellular adhesion molecule 1; *Itga5*: Integrin, alpha subunit 5; *Itgb3*: Integrin, beta subunit 3; *Il6*: Interleukin 6; *Jag1*: Jagged-1; *Loxl3*: Lox-like protein 3; *Mmp3*: matrix metallopeptidase 3; *Pcolce2*: Procollagen C-endopeptidase enhancer; *Sod2*: superoxide dismutase 2; *TGFß1*: Transforming growth factor beta 1; *Tnfα*: Tumor necrosis factor alpha; *Tagln*: Transgelin; *Timp1*: Tissue inhibitor of metalloproteinases 1; *Tfpi2*: Tissue factor pathway inhibitor.

Protein Identification Using LC-MS/MS

Sample preparation

Samples were prepared using a modified enhanced Filter Aided Sample Preparation (eFASP) in order to increase proteome coverage and sample recovery for quantitative proteomic experiments. Before their use, 0.5 mL Amicon® ultra centrifugal filters equipped with a cut-off of 10 kDa (EMD Millipore, Darmstadt, Germany) were incubated overnight with a passivation solution containing 5% (v/v) Tween®-20, and rinsed, then, with ultrapure water. 100 µg of protein were transferred to an Amicon® filter, followed by 100 µL of exchange buffer (8 M urea, 0.2% DCA, 100 mM ammonium bicarbonate pH 8.8). After a centrifugation step of 30 min at 10,000 g, the filtrate was removed. 200 µL of exchange buffer were next added to the Amicon® filter, which was again centrifuged. This operation was repeated twice. The proteins were alkylated for 1 h at room temperature (20°C) in the dark using 100 µL of alkylation buffer (8 M urea, 50 mM iodoacetamide, and 100 mM ammonium bicarbonate, pH 8.8). The Amicon® filter was centrifuged again for 30 min at 10,000 g and the filtrate discarded. After this alkylation step, 200 µL of exchange buffer were added to the Amicon® filter, which was again centrifuged for 30 min at 10,000 g, and the filtrate discarded. 200 µL of digestion buffer (0.2% DCA, 50 mM ammonium bicarbonate pH 8.8) were added to the Amicon® filter, before another centrifugation step (30 min at 10,000 g). This operation was repeated twice, the filtrate being removed and discarded. The Amicon® filter was transferred to a new 2 mL concentrator collection tube. 100 µL of digestion buffer with 40 µL of trypsin/LysC (Promega, Madison, USA) were added and incubated in the Amicon® filter while shaking in a heating block tube (MHR23, Hettich, Netherlands) overnight at 37°C. Thereafter, the peptides present in the Amicon® filter were recovered in the tube by centrifugation for 15 min at 10,000 g. To maximize the peptide recovery, two washing steps were implemented with 50 µL of ammonium bicarbonate solution (50 mM pH 8.8). The filtrate containing all peptides was next transferred to a 1.5 mL Eppendorf® microtube (Eppendorf, Hamburg, Germany). 200 µL of ethyl acetate with 2.5 µL of TFA were added, causing the peptide precipitation (white color). At once, 800 µL of ethyl acetate were added again, the resulting solution centrifuged for 10 min at 10,000 g and the organic phase eliminated. This operation was repeated twice. The Eppendorf® microtube was placed for 5 min at 60°C in a heating block (SBH130, Stuart, Staffordshire, UK) to let the remaining ethyl acetate evaporate. The samples were dried at room temperature in a SpeedVac™ Concentrator (EppendorfTM Concentrator Plus, Eppendorf). Next, 100 µL of a methanol/water (50/50) mixture were added to the resulting solid phase and let to evaporate. For Mass Spectrometry (MS) analysis, the samples were dissolved in 10 µL of ultrapure water supplemented with 0.1% of formic acid. The sample concentration was estimated by measuring the optical density (OD) at 215 nm of 1 µL of the solution using a droplet UV spectrometer (DS-11+, Denovix, Wilmington, USA). Finally, the concentration of

the sample was adjusted to 1 µg/µL by dilution with ultrapure water containing 0.1% formic acid (FA) before analysis. Each sample were analyzed in triplicate.

LC-MS/MS Orbitrap eFASP

LC-MS/MS protein analysis was performed on an Orbitrap Q Exactive plus Mass Spectrometer hyphenated to a U3000 RSLC Microfluidic HPLC System (ThermoFisher Scientific). 1 µL of the peptide mixture at a concentration of 1 µg/µL was injected with a solution A (5% v/v acetonitrile and 0.1% formic acid) for 3 min at a flow rate of 5 µL/min on an Acclaim PepMap100 C18 pre-column (5 µm, 300 µm i.d.×5 mm) (ThermoFisher Scientific). The peptides were next separated on a C18 Acclaim PepMap100 C18 reversed phase column (3 µm, 75 mm i.d. × 500 mm) (ThermoFisher Scientific), using a linear gradient (5-40%) of solution B (75% ACN and 0.1% formic acid) using a flow-rate of 250 mL/min in 160 min followed by 100% solution B for 5 min. The column was regenerated by washing it for 5 min with solution B and then re-equilibrated with solution A during 10 min. The column and the pre-column were placed in an oven at a temperature of 45°C. The total duration of the analysis was 180 min. The LC (liquid chromatography) runs were acquired in positive ion mode with MS scans from m/z 350 to 1,500 in the Orbitrap mass analyzer with a 70,000 resolution for MS and 35,000 resolution for MS/MS. The automatic gain control was set at 1×106 for MS and the ion injection time is 100 ms. MS/MS scans were sequentially acquired in the high-energy collision dissociation cell for the 10 most-intense ions detected in the full MS survey scan. Automatic gain control was set at 5×105 and the ion injection time is 160 ms, and the normalized collision energy was set to 28 eV. Dynamic exclusion was set at 90 s and ions with 1 and more than 8 charges were excluded.

SUPPLEMENTARY RESULTS

Table 1

sheet 1. Enrichment analysis of TGFβ1_up^g using online tool Metascape (excel file). Spreadsheet “annotation”: annotations of TGFß1_up^g. Spreadsheet “enrichment”: details of the enrichment analysis using Metascape software as described in Methods (http://metascape.org/gp/index.html#/main/step1). Terms, log(*p-value*), log(*q-value*), and ratio of genes (detected/all members of term) are detailed.

sheet 2. Enrichment analysis of TGFβ1_down^g using online tool Metascape (excel file). Spreadsheet “annotation”: annotations of TGFß1_down^g. Spreadsheet “enrichment”: details of the enrichment analysis using Metascape software as described in Methods. Terms, log(*p-value*), log(*q-value*), and ratio of genes (detected/all members of term) are detailed.

sheet 3. Enrichment analysis of TNFα_up^g using online tool Metascape (excel file). Spreadsheet “annotation”: annotations of TNFα_up^g. Spreadsheet “enrichment”: details of the enrichment analysis using Metascape software as described in Methods. Terms, log(*p-value*), log(*q-value*), and ratio of genes (detected/all members of term) are detailed.

sheet 4. Enrichment analysis of TNFα_down^g using online tool Metascape (excel file). Spreadsheet “annotation”: annotations of TNFα_down^g. Spreadsheet “enrichment”: details of the enrichment analysis using Metascape software as described in Methods. Terms, log(*p-value*), log(*q-value*), and ratio of genes (detected/all members of term) are detailed.

Table 2

sheet 1. Enrichment analysis of TGFβ1_up^p using online tool Metascape (excel file). Spreadsheet “annotation”: annotations of TGFß1_up^p. Spreadsheet “enrichment”: details of the enrichment analysis using Metascape software as described in Methods (http://metascape.org/gp/index.html#/main/step1). Terms, log(*p-value*), log(*q-value*), and ratio of proteins (detected/all members of term) are detailed.

sheet 2. Enrichment analysis of TGFβ1_down^p using online tool Metascape (excel file). Spreadsheet “annotation”: annotations of TGFß1_down^p. Spreadsheet “enrichment”: details of the enrichment analysis using Metascape software as described in Methods. Terms, log(*p-value*), log(*q-value*), and ratio of proteins (detected/all members of term) are detailed.

sheet 3. Enrichment analysis of TNFα_up^p using online tool Metascape (excel file). Spreadsheet “annotation”: annotations of TNFα_up^p. Spreadsheet “enrichment”: details of the enrichment analysis using Metascape software as described in Methods. Terms, log(*p-value*), log(*q-value*), and ratio of proteins (detected/all members of term) are detailed.

sheet 4. Enrichment analysis of TNFα_down^p using online tool Metascape (excel file). Spreadsheet “annotation”: annotations of TNFα_down^p. Spreadsheet “enrichment”: details of the enrichment analysis using Metascape software as described in Methods. Terms, log(*p-value*), log(*q-value*), and ratio of proteins (detected/all members of term) are detailed.

Supplementary Figure S1.

Gene expression assessed by RT-qPCR in TNFα- and TGFß1-treated MEFs compared to control MEFs. Numbers are expressed as median fold change (IQR) compared to control MEFs after a 6-h, 12-h, 18-h, and 24-h stimulation. At least n=12 for each condition from 3 independent experiments. We observed that the profibrotic profile induced by TGFß1 is more marked after a 24-h stimulation. Moreover, the distinctiveness between TNFα- and TGFß1-treated MEFs was more pronounced after a 24-h stimulation. P-value: **** ≤ 0.001, *** ≤ 0.001, ** ≤ 0.01, * ≤ 0.05. ns: > 0.05.

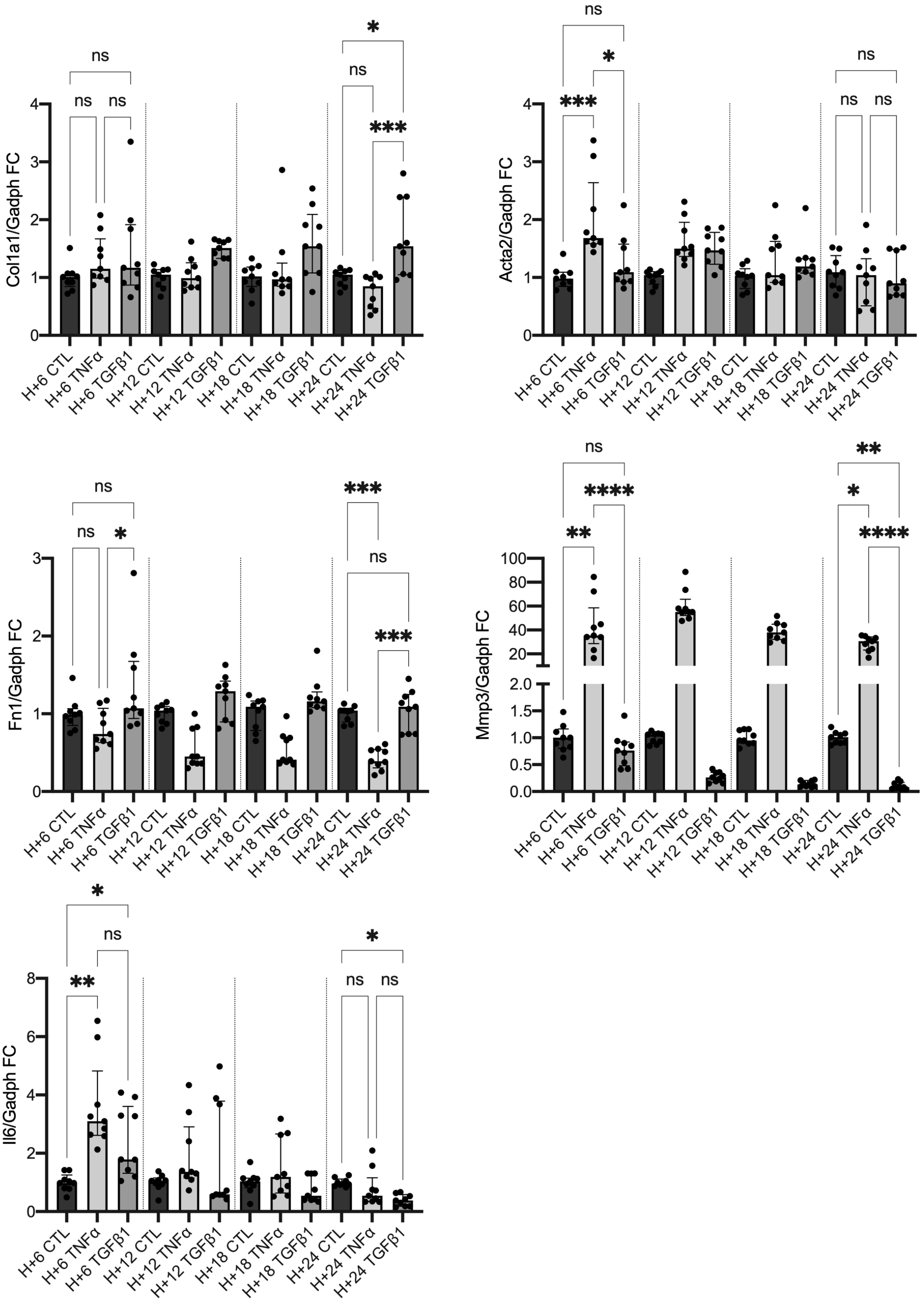

Supplementary Figure S2. Transcript expression profiles of control, TGFß1-treated and TNFα-treated MEFs using RNA microarray.

(a) Number of DEGs in MEFs treated by TGFß1 or by TNFα respectively compared to control MEFs (*q-value* <0.05) according to the absolute fold change thresholds. (b-c) Number of DEGs (*q* <0.05) in MEFs treated by TGFß1 (b) or TNFα (c) compared to control MEFs according to the way of deregulation (up or down) and to the absolute fold change threshold. (d) Principal component analysis of MEFs (controls, treated by TGFß1 or TNFα) assessed by RNA microarray using R software (version R 3.6.0,) (https://www.r-project.org). N=3 biological replicas (3 independent experiments).

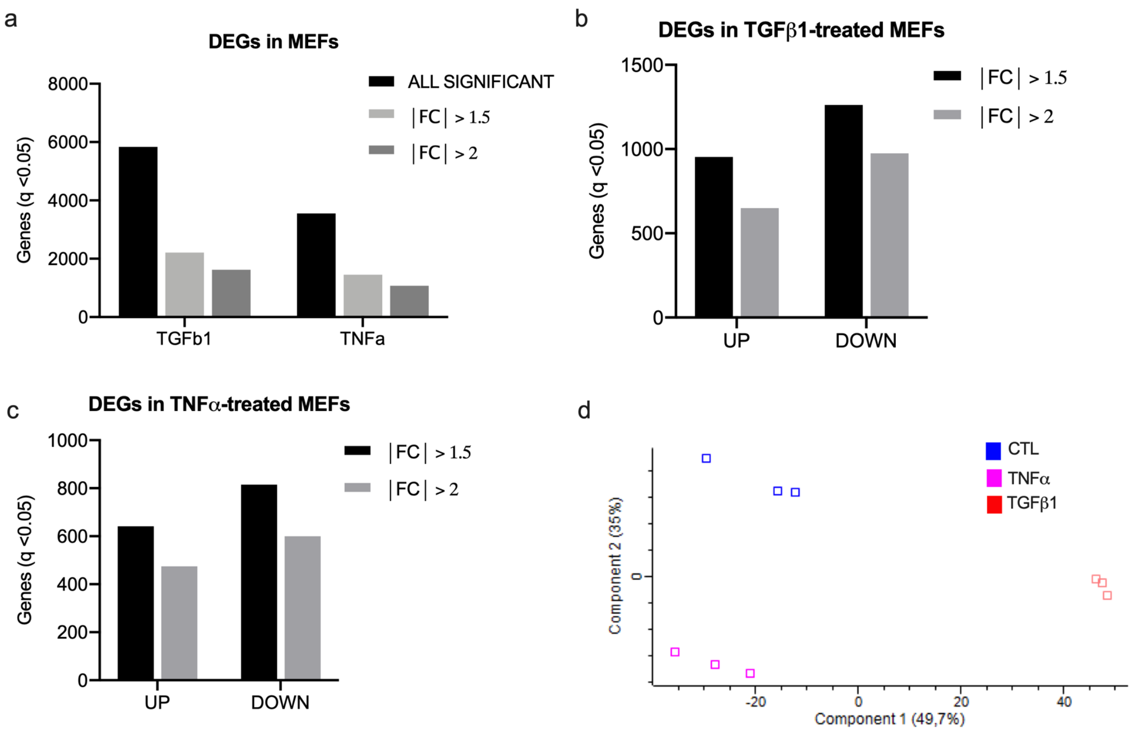

Supplementary Figure S3. Enrichment analysis of the clusters TGFß1_up^{genes} and TNFα_up^{genes}.

All significantly enriched terms in TGFß1_up^{genes} (a) and TNFα_up^{genes} (c) were hierarchically clustered into a tree based on Kappa-statistical similarities among their gene memberships. A subset of representative terms from theses clusters have been converted into a network layout. Each term is represented by a circle node, whose size is proportional to the number of input genes associated to that term, and whose color represents a cluster identity. Terms with a similarity score > 0.3 are linked by an edge. Each cluster was named according to the terms memberships. (b, d) The same enrichment network with nodes colored following a p-value order. The enrichment analysis was performed using Metascape (http://metascape.org/gp/index.html - /main/step1).

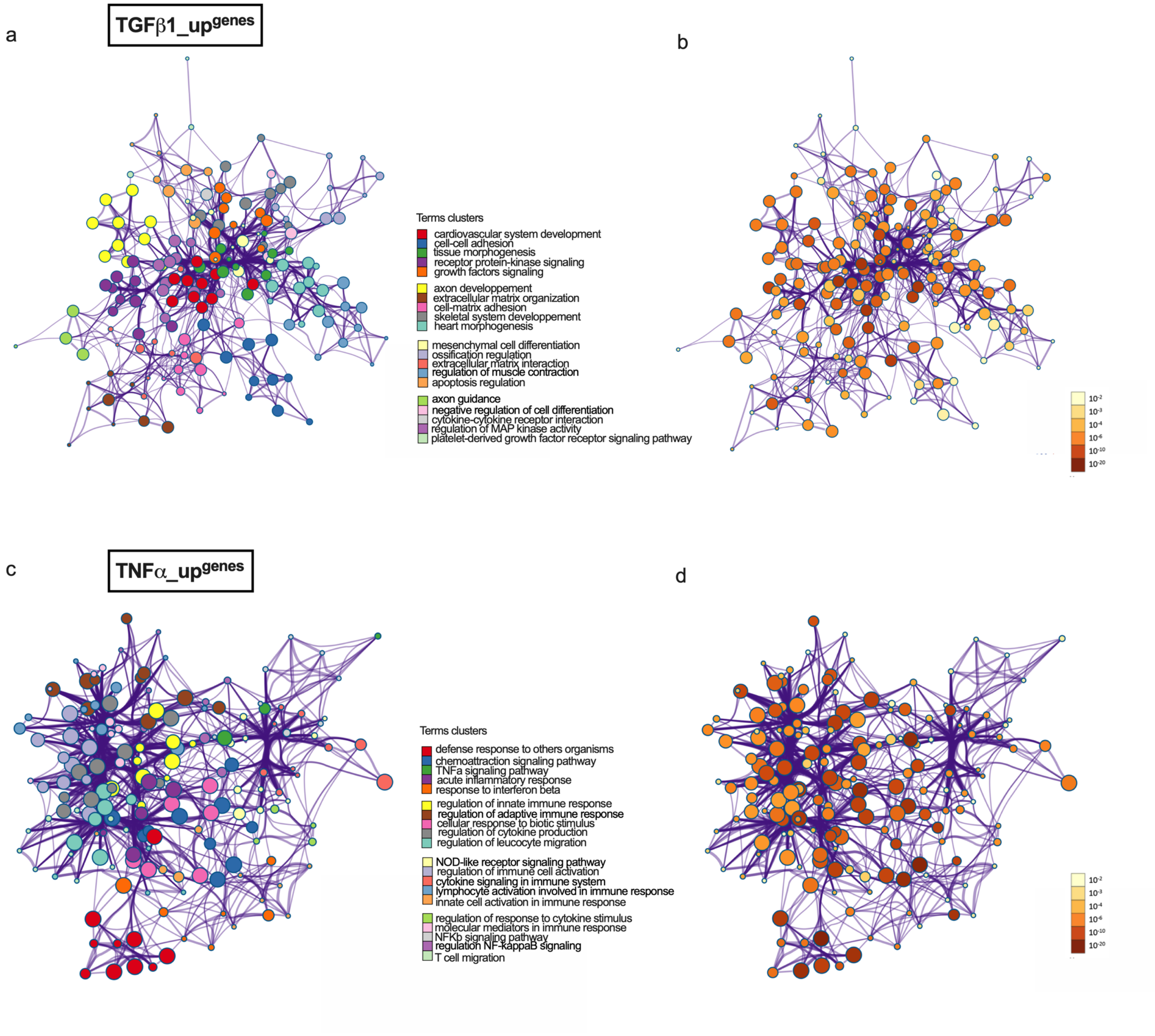

Supplementary Figure S4. Proteome analysis of control, TGFß1- and TNFα-treated MEFs using LC-MS/MS.

(a) Number of DEPs in MEFs treated by TGFß1 or by TNFα compared to control MEFs. (b) Number of DEPs (q <0.05) in MEFs treated by TGFß1 or TNFα compared to control MEFs according to the way of deregulation (up or down). (c) Principal component analysis of MEFs (controls, treated by TGFß1 or TNFα) assessed by LC-MS/MS generated using R software (version R 3.6.0,) (https://www.r-project.org). (d-e) Venny diagrams summarizing all DEGs and DEPs (q-*value* < 0.05) in TGFß1- and TNFα-treated MEFs compared to control MEFs using Venny[2.1] (https://bioinfogp.cnb.csic.es/tools/venny). N=3 biological replicas (3 independent experiments).

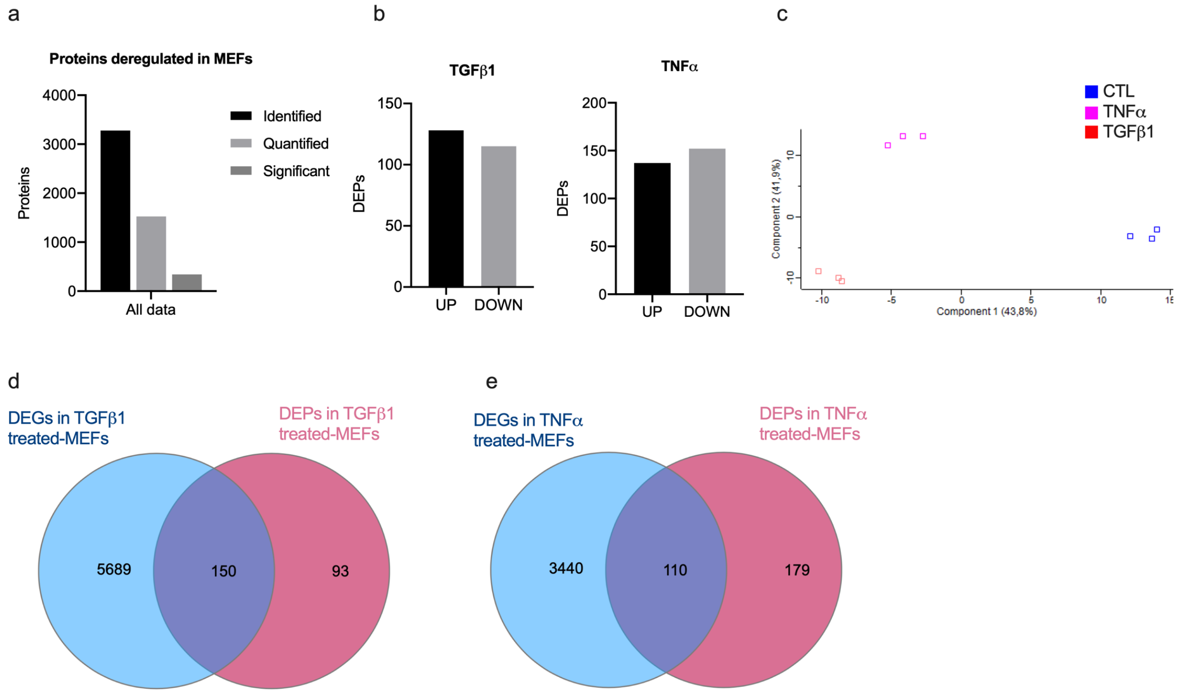

Supplementary Figure S5. Gene expression ECM-organization and inflammatory response pathway gene sets in control, TGFß1- and TNFα-treated MEFs.

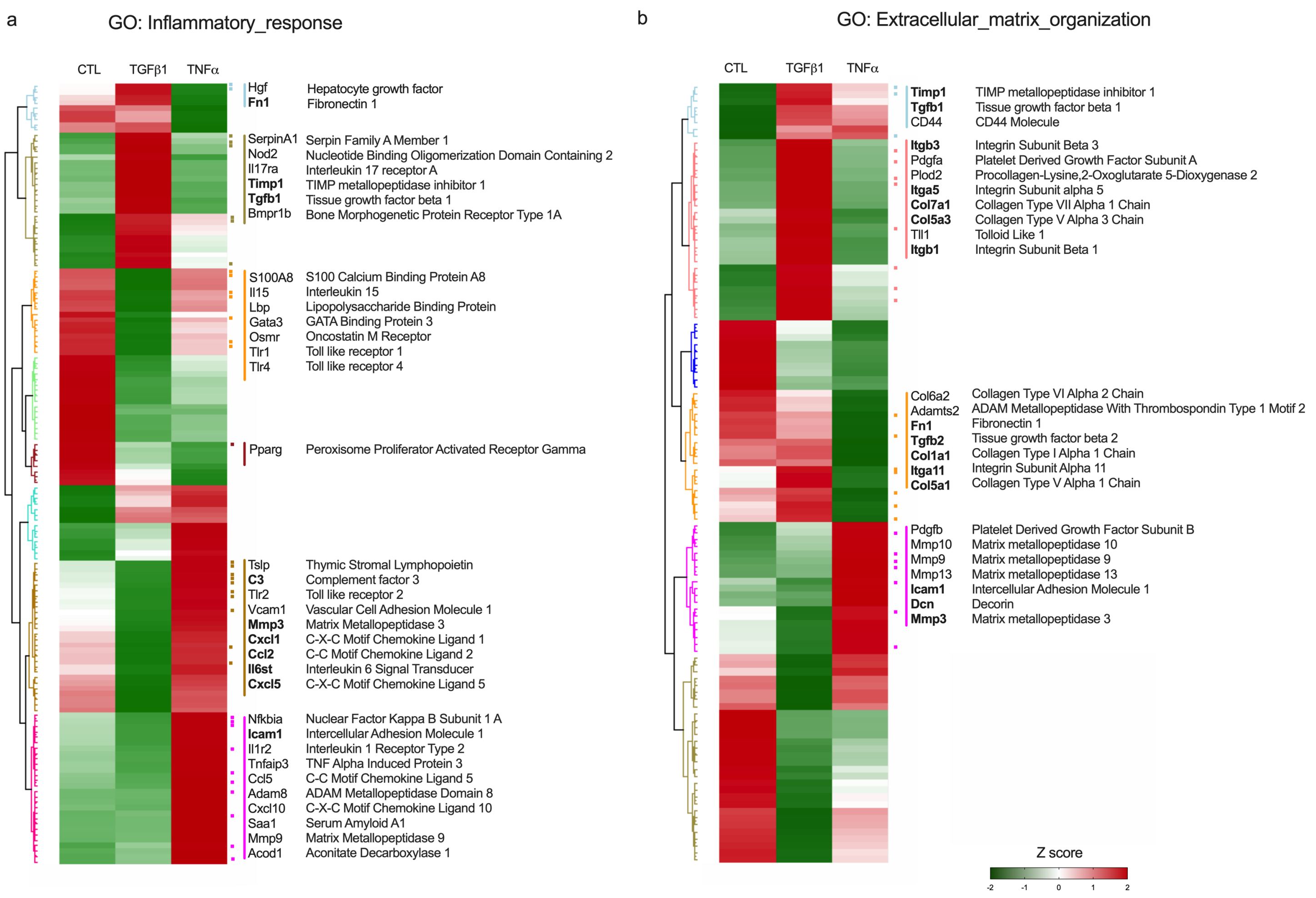

(a) Gene expression associated with ECM organization pathway gene set in controls, TGFß1- and TNFα-treated MEFs assessed by RNA microarray. (b) Gene expression associated with inflammatory response pathway gene set in controls, TGFß1- and TNFα-treated MEFs assessed by RNA microarray. Gene lists come from GSEA database. Only genes with significant expression variations are presented in heatmaps (q-value < 0.05 and at least fold-change > 2 compared to controls). Heatmaps and hierarchical clustering were performed using Perseus software (version 1.60.2) (https://maxquant.net/perseus/). Genes are marked and mentioned from top to bottom (square) and according to the membership of row clustering (colored bar).

Supplementary Figure S6. Gene set expression assessed by RT-qPCR in TNFα- and TGFß1-treated MEFs compared to control MEFs.

Numbers are expressed as median fold change (IQR) compared to control MEFs at 24h of stimulation. At least n=4 for each condition from 2 independent experiments. P-value: **** ≤ 0.001, *** ≤ 0.001, ** ≤ 0.01, * ≤ 0.05. ns: > 0.05.

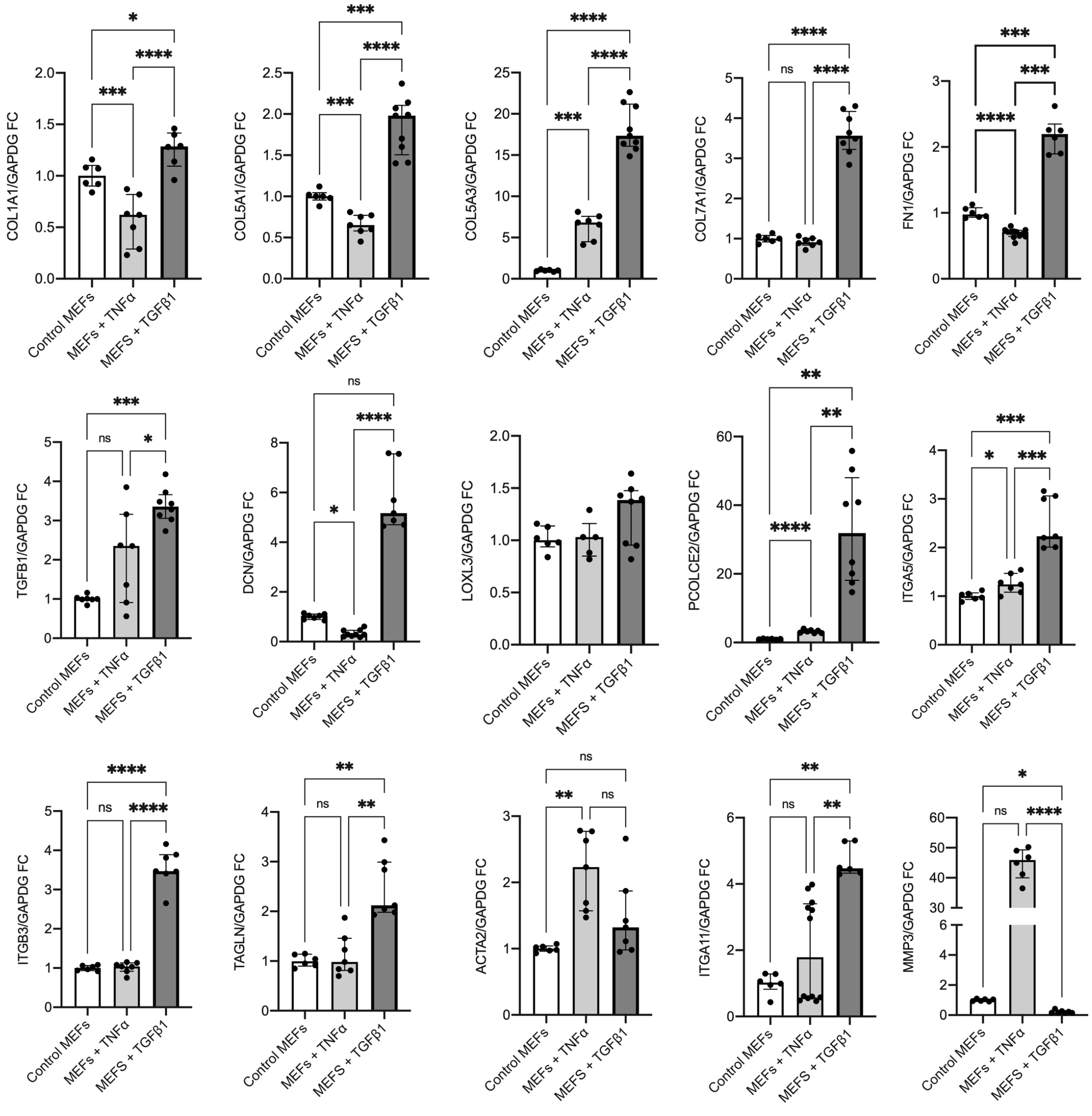

Supplementary Figure S7. Gene set expression assessed by RT-qPCR in TNFα- and TGFß1-treated MEFs compared to control MEFs.

Numbers are expressed as median fold change (IQR) compared to control MEFs at 24h of stimulation. At least n=4 for each condition from 2 independent experiments. P-value: **** ≤ 0.001, *** ≤ 0.001, ** ≤ 0.01, * ≤ 0.05. ns: > 0.05.

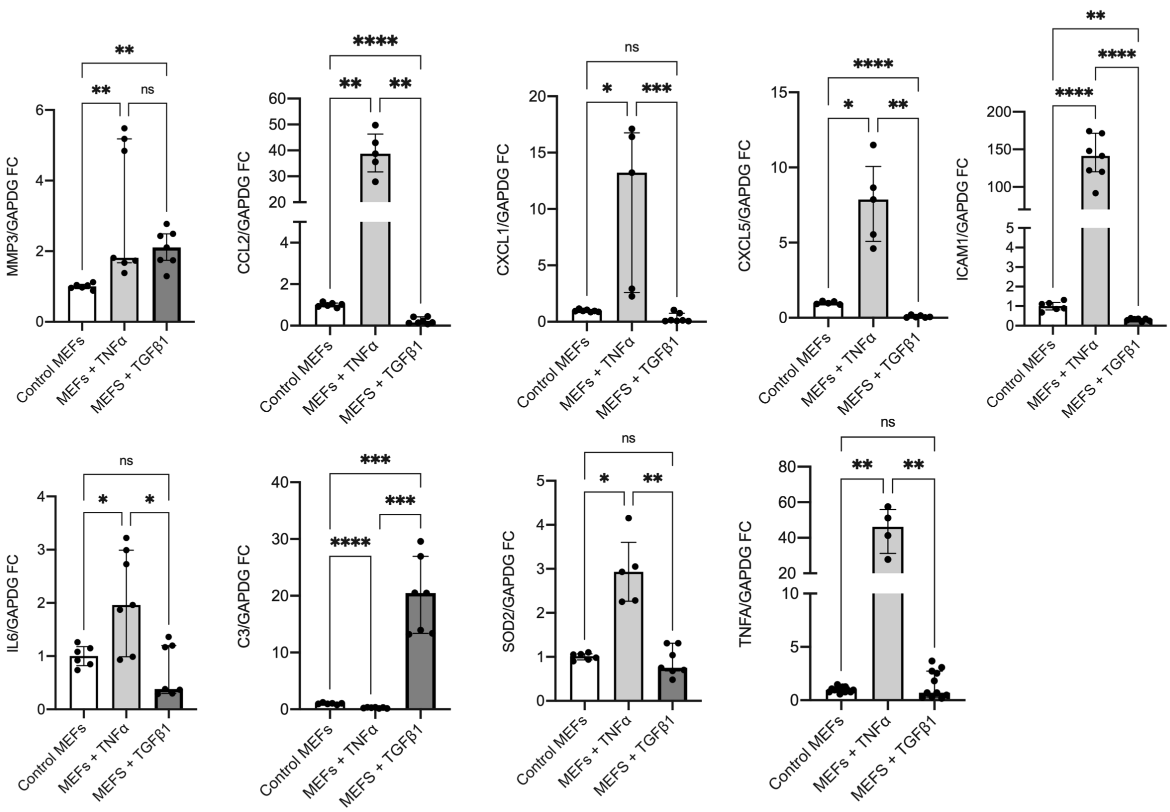

Supplementary Figure S8. Uncropped images of Fig. 5e.

Full unedited western blots. The cropped area corresponding to Fig. 5e in the manuscript is shown by a red frame.

anti-Col1a1

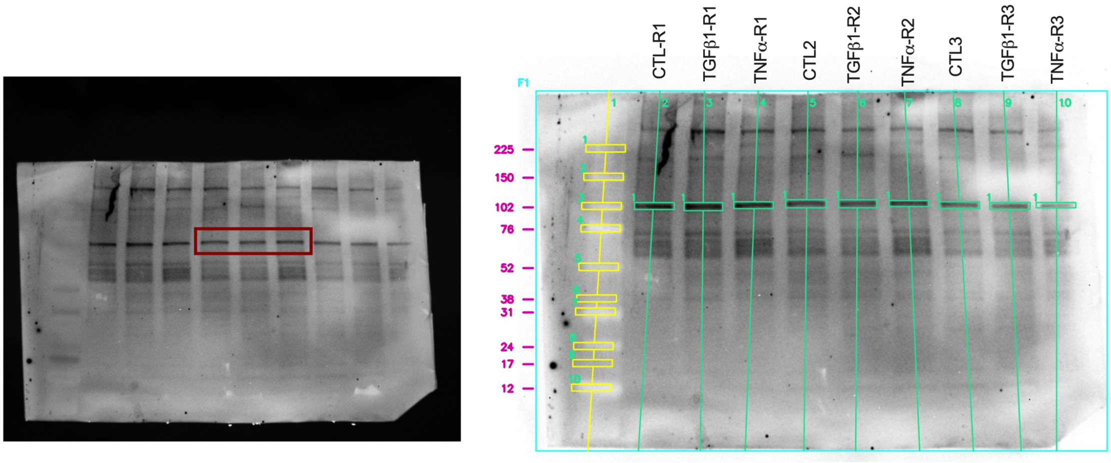

anti-α-SMA

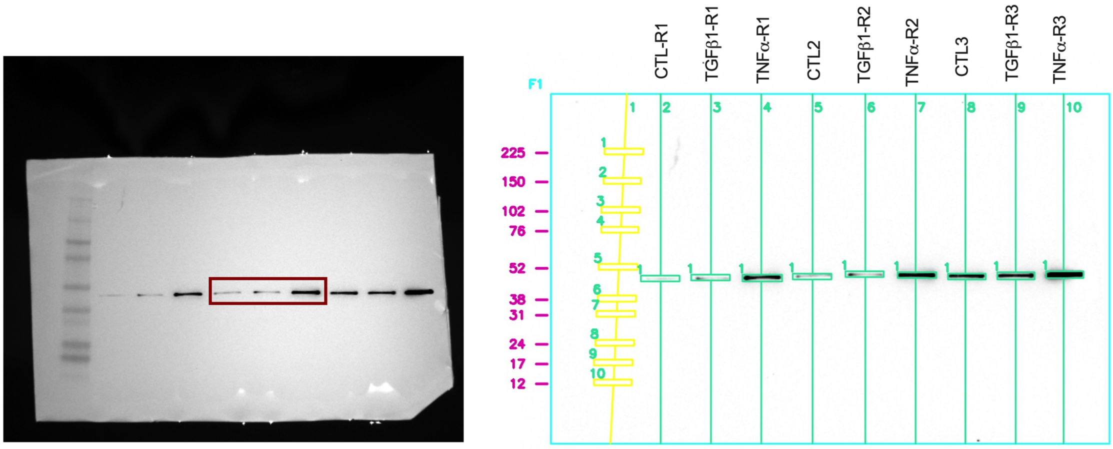

anti-β-actin

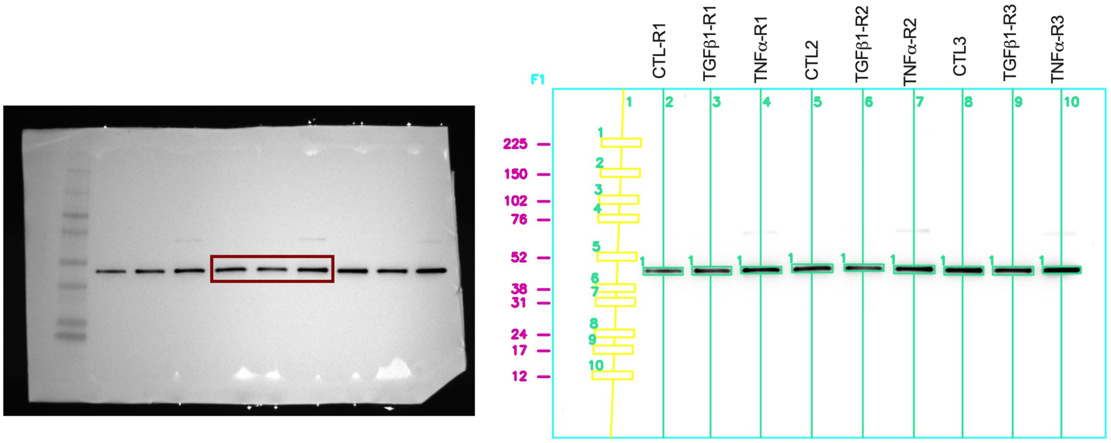

Supplementary Figure S9. Uncropped images of Fig. 5g.

Full unedited western blots. The cropped area corresponding to Fig. 5g in the manuscript is shown by a red frame.

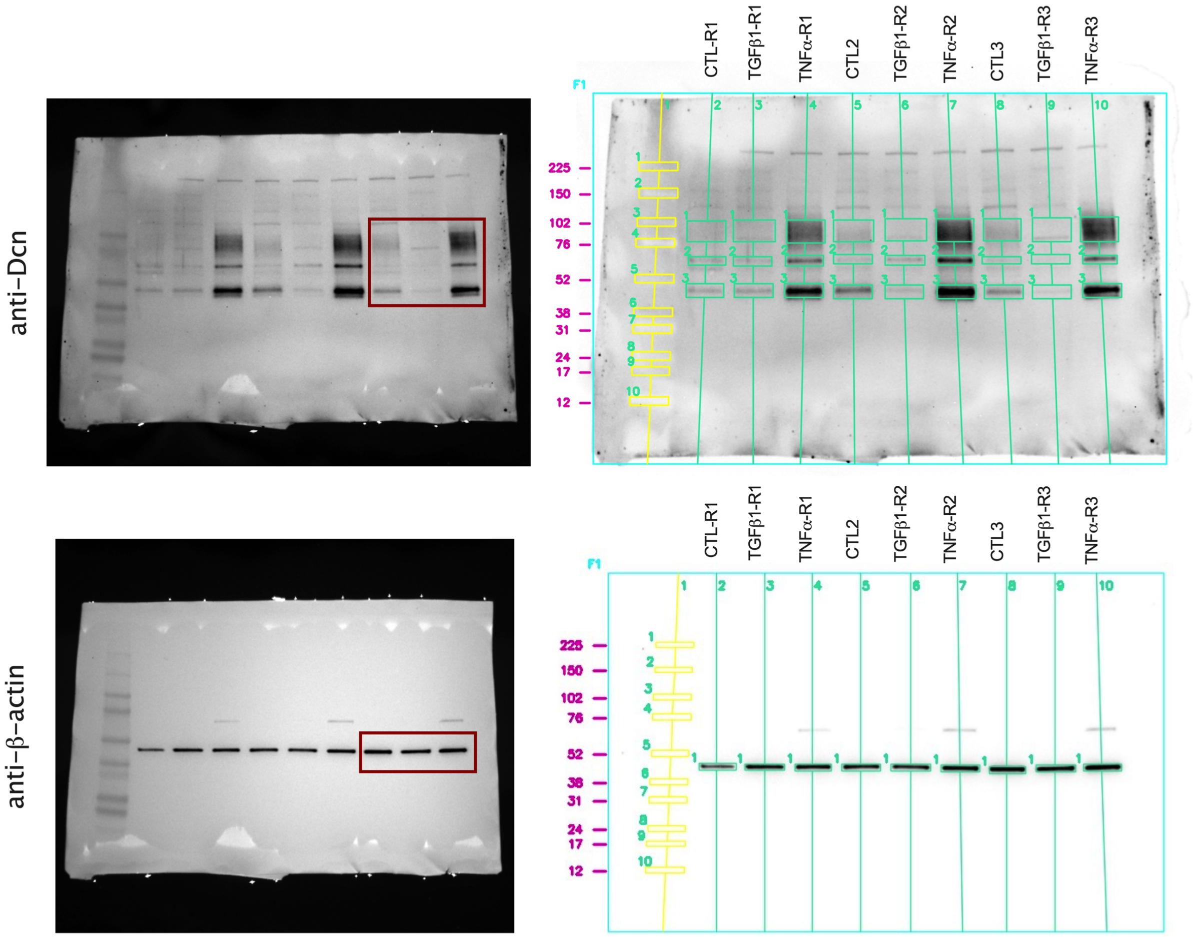

Supplementary Figure S10. Uncropped images of Fig. 5f.

Full unedited western blots. The cropped area corresponding to Fig. 5f in the manuscript is shown by a red frame.

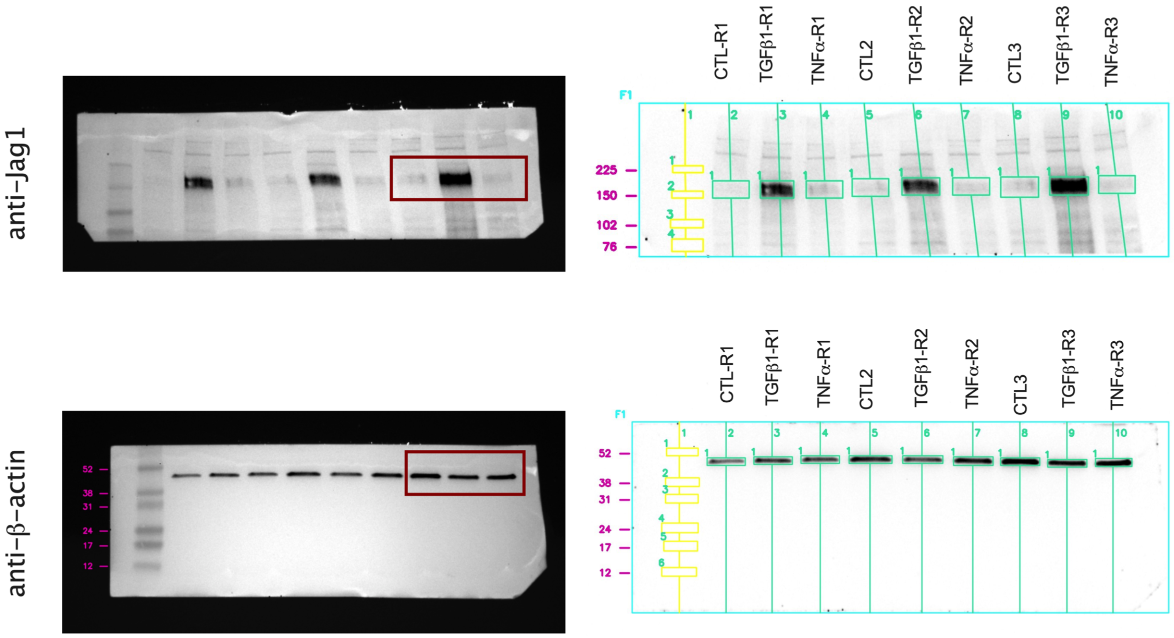

Supplementary Figure S11. Gene set expression assessed by RT-qPCR in HOCL MPFs and PBS MPFs.

Numbers are expressed as median fold change (IQR) compared to PBS MPFs. At least n=4 for each condition from one independent experiment. P-value: **** ≤0.001, *** ≤0.001, ** ≤0.01, * ≤0.05. ns: >0.05.

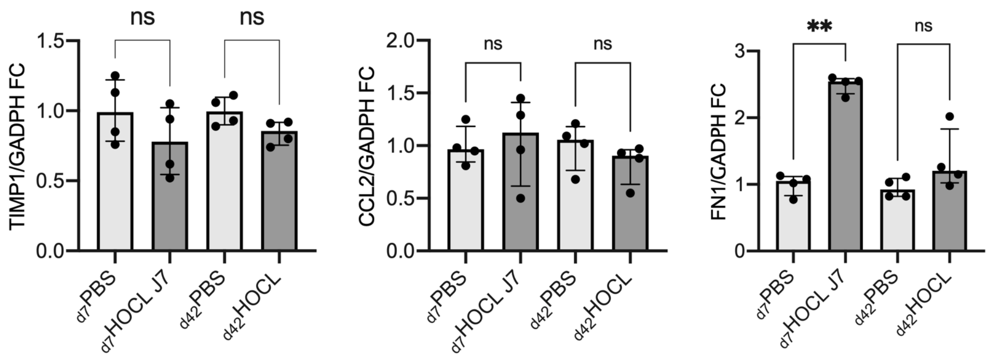

Supplement: Supplementary file 1 — Supplementary Information 1. [file 41598_2022_15640_MOESM1_ESM.pdf]
